# Tensile Strength Estimation of UHPFRC Based on Predicted Cracking Location Using Deep Learning

**DOI:** 10.3390/ma18102237

**Published:** 2025-05-12

**Authors:** Xin Luo, Takashi Matsumoto

**Affiliations:** 1 Graduate School of Engineering, Hokkaido University, Sapporo 060-8628, Japan; xin.luo.a1@elms.hokudai.ac.jp; 2 Faculty of Public Policy, Hokkaido University, Sapporo 060-0810, Japan

**Keywords:** ultra-high-performance fiber-reinforced concrete (UHPFRC), tensile strength estimation, fiber count, deep learning, X-ray computed tomography (CT) analysis

## Abstract

Ultra-high-performance fiber-reinforced concrete (UHPFRC) exhibits exceptional tensile properties, but its tensile strength is highly dependent on fiber distribution, orientation, and count, making accurate strength estimation challenging. This study introduces a novel approach in which tensile strength estimation is achieved by analyzing fiber characteristics at predicted cracking locations using deep learning. Using X-ray computed tomography (CT) and image analysis techniques, the fiber orientation factor (μ_0_) and average efficiency factor ((μ_1_)^−^) were determined at predicted cracking locations. A deep learning model (YOLOv11) was trained to identify regions with a defective distribution, achieving a mean Average Precision (mAP@0.5) of 0.87, demonstrating its high reliability in predicting cracking locations. The overall cracking location prediction success rate was 73% for strain-hardening specimens. The estimated tensile strength was then compared with uniaxial tensile test (UTT) results, revealing an average experiment-estimation error of 5.72% and an average theory-estimation error of 3.34% for strain-hardening specimens, whereas strain-softening specimens exhibited significantly higher errors, with an average experiment-estimation error of 43.09% and an average theory-estimation error of 15.73%. These findings highlight the strong correlation between fiber count, cracking behavior, and tensile strength in UHPFRC, offering a trustworthy, non-destructive framework for estimating tensile performance in UHPFRC elements.

## 1. Introduction

Ultra-high-performance fiber-reinforced concrete (UHPFRC) has emerged as an industry-standard material in modern construction due to its superior tensile performance and durability [1,2]. Unlike conventional concrete, UHPFRC exhibits strain-hardening behavior under tensile stress, characterized by the formation of multiple microcracks while maintaining its tensile load-carrying capacity [3,4]. This unique property is attributed to the synergistic interaction between its densely packed cementitious matrix and dispersed steel fibers, which bridge cracks and enhance tensile resistance [5,6]. These exceptional qualities make UHPFRC indispensable for critical infrastructure projects, such as bridges, high-rise buildings, and tunnels, where tensile strength is crucial for ensuring structural safety and durability [7,8]. In addition to new construction applications, UHPFRC has also been increasingly used for the retrofitting and strengthening of existing reinforced concrete (RC) structures, owing to its superior tensile strength and durability. Recent studies have demonstrated its effectiveness in enhancing the load-carrying capacity and seismic resilience of RC components [9,10,11]. Building on these findings, recent developments have further expanded the application of UHPFRC to prefabricated structural elements, seismic retrofit systems, and energy-absorbing components in bridges and tunnels, underscoring the critical need for accurately predicting its tensile performance under various loading scenarios [12,13]. In pursuit of improved in situ performance reliability, particular attention has been given to the control of fiber orientation during fabrication, which has been shown to play a pivotal role [14,15].

Nevertheless, the tensile strength of UHPFRC remains significantly influenced by the distribution, orientation, and count of fibers, leading to inherent variability and challenges in ensuring consistent mechanical behavior [16,17]. More recently, quantitative investigations have revealed that even minor deviations in local fiber orientation can result in considerable scatter in tensile performance, especially under flexural and tensile loading conditions [18].

This variability has driven ongoing efforts to reliably estimate tensile strength, as accurate assessment is essential for optimizing material performance and guiding structural design [19,20]. Early attempts at tensile strength estimation relied heavily on empirical methods, which, while reliable, were time-consuming and resource intensive [21,22]. To address these limitations, researchers have developed theoretical models, progressively refining them to better account for fiber contributions and their interactions with the matrix [23]. This evolution has advanced tensile strength estimation from simplistic assumptions to sophisticated frameworks incorporating the distribution, orientation, and count of fibers [24,25].

The earliest estimation model, exemplified by Formula (1) in Table 1, was based on traditional concrete theories, treating materials as isotropic and homogeneous. These models related tensile strength (*f*_*t*_) to compressive strength (*f*_*c*_) using a constant (*α*) to represent the proportional relationship. While adequate for plain concrete, such models failed to account for fiber reinforcement, leading to significant underestimations of the tensile capacity of UHPFRC [25,26,27]. To address this, researchers introduced fiber-related parameters such as volume fraction (*V*_*f*_) and interfacial bond strength (*τ_f_*), as shown in Formula (2) [3,25,28]. These models acknowledged the influence of fibers on tensile performance but assumed a uniform distribution, which limited their applicability in real-world scenarios where fiber alignment is non-uniform [14,29]. The introduction of the fiber orientation factor (*μ*_0_), represented in Formula (3), marked a critical step forward. By quantifying the alignment of fibers relative to the direction of tensile loading, this model accounted for anisotropic behavior, significantly improving estimation accuracy. Building on this, efficiency factor (*μ*_1_) was incorporated in Formula (4) to represent fiber pull-out resistance and matrix-fiber interaction efficiency, resulting in a more comprehensive framework for tensile strength estimation [30,31]. Subsequently, the SIA 2052-2016 [32] guidelines formalized these advancements into Formula (5), which was derived from the analysis of cracking location cross-sections. This formula incorporated the fiber aspect ratio (*l_f_*/*d_f_*), providing a localized perspective on fiber contributions. By focusing on critical cracking locations, this approach offers a viable pathway for estimating UHPFRC tensile performance, though it does not represent a fully standardized solution and still exhibits certain limitations. While these models effectively captured global fiber behavior, recent studies have highlighted the critical role of local fiber distribution within cracking locations in accurately estimating tensile strength. Shen et al. [33] introduced a uniformity factor (*μ*_2_) into Formula (6), addressing the heterogeneity of fiber distribution at fracture-critical zones. Their research demonstrated that an uneven distribution within the area increases susceptibility to crack initiation, underscoring the need to consider localized characteristics. Furthermore, the parameter λ = *V_f_μ*_0_*μ*_1_ was introduced here as a scalar description of local fiber distribution for each measured region in UHPFRC with respect to the tensile loading direction. This parameter demonstrates a strong correlation between fiber count and cracking locations, highlighting the significant influence of fiber count in critical zones on tensile performance and failure mechanisms.

For λ = *V_f_μ*_0_*μ*_1_, where *V_f_* remains constant, *μ*_0_ demonstrates a positive correlation with the fiber count and also demonstrates a positive relationship with *μ*_1_. Consequently, *λ* is directly proportional to the fiber count. In regions of a UHPFRC specimen with higher fiber counts, *λ* increases, reducing the probability of these regions becoming cracking locations. Conversely, in regions with lower fiber counts, *λ* decreases, increasing the probability of fracture occurrence in these regions. By accurately identifying potential cracking locations in UHPFRC specimens, it becomes feasible to estimate their tensile strength using Formula (5), which incorporates key fiber parameters to provide a robust framework for estimating tensile strength.

Building on this principle, the objective of this study is to estimate the tensile strength of UHPFRC using the widely used Formula (5). To preclude the homogenization of fiber distributions, orientation and, consequently, similar fiber counts that may occur with a single casting method, four different casting methods were employed to achieve varied fiber distribution characteristics in UHPFRC specimens. First, the fiber distribution characteristics of dog-bone-shaped specimens were analyzed using X-ray CT scanning and image analysis software, Fiji [34]. Next, cracking locations were predicted using a deep learning-based approach. Finally, the tensile strength of the UHPFRC specimens was estimated using *μ*_0_ and $\bar{\mu_{1}}$ derived from the predicted cracking locations. Additionally, the tensile strength estimated at the cracking locations using the proposed method was compared with the tensile strength obtained from uniaxial tensile tests, demonstrating the effectiveness and reliability of the approach.

However, while previous models, such as those proposed by Wille et al. [23,24] and Shen et al. [33], have progressively refined tensile strength estimation by introducing fiber orientation (*μ*₀), efficiency ($\bar{\mu_{1}}$), and uniformity factors (*μ*_2_), these methods are fundamentally limited by their reliance on post-failure section analysis. They typically assume that cracking locations are known and assess fiber characteristics at these locations after mechanical testing. Consequently, they are less suitable for non-destructive evaluation and real-time quality control applications. Furthermore, most existing approaches neglect the spatial variability induced by non-uniform fiber distribution during casting, thereby limiting their predictive accuracy for actual structural elements.

To overcome these limitations, this study proposes a novel integrated framework that predicts potential cracking locations using a deep learning-based object detection model and subsequently analyzes local fiber characteristics via CT image processing. This approach enables pre-failure, non-destructive tensile strength estimation, offering significant improvements in both practical applicability and estimation accuracy compared to traditional post hoc methods.

**Table 1:** Evolution of estimation formulas for UHPFRC tensile strength.

Formula No.	Formula	Description	Key Improvements	Reference
(1)	*f_t_* = *αf_c_*	Relates tensile strength (*f_t_*) to compressive strength (*f_c_*), ignoring fiber effects.	Simplistic model; assumes isotropy and homogeneity; fails to account for fiber reinforcement.	Traditional concrete mechanics.
(2)	*f_t_* = *f_c_* + *kV_f_τ_f_*	Incorporates fiber volume fraction (*V_f_*) and interfacial bond strength (*τ_f_*); assumes uniform fiber distribution.	Recognizes fiber contributions; initial step towards fiber-reinforced models.	Hannant (1978) [35]; Naaman (1972) [36].
(3)	*f_t_ = μ*_0_*V_f_τ_f_*	Introduces fiber orientation factor (*μ*_0_) to account for anisotropic alignment.	Captures directional fiber alignment in tensile loading; improves accuracy for anisotropic materials.	Wille et al. (2011) [3].
(4)	*f_t_* = *μ*_0_*μ*_1_*τ_f_V_f_*	Adds efficiency factor (*μ*_1_) for fiber pull-out resistance and fiber-matrix interactions.	Enhances accuracy by accounting for pull-out effects and improved fiber-matrix representation.	Wille et al. (2014) [5].
(5)	*f_t_* = *μ*_0_*μ*_1_*τ_f_V_f_d_f_*/*l_f_*	Adds fiber aspect ratio (*l_f_*/*d_f_*).	Standardized for practical applications in UHPFRC design and engineering.	SIA 2052-2016 [32].
(6)	*f_t_* = *μ*_0_*μ*_1_*μ*_2_*τ_f_V_f_d_f_*/*l_f_*	Incorporates uniformity factor (*μ*_2_) to address localized fiber distributions.	Enhances accuracy by accounting for local fiber distributions.	Shen et al. (2020) [33].

## 2. Materials and Methods

**Figure 1:**
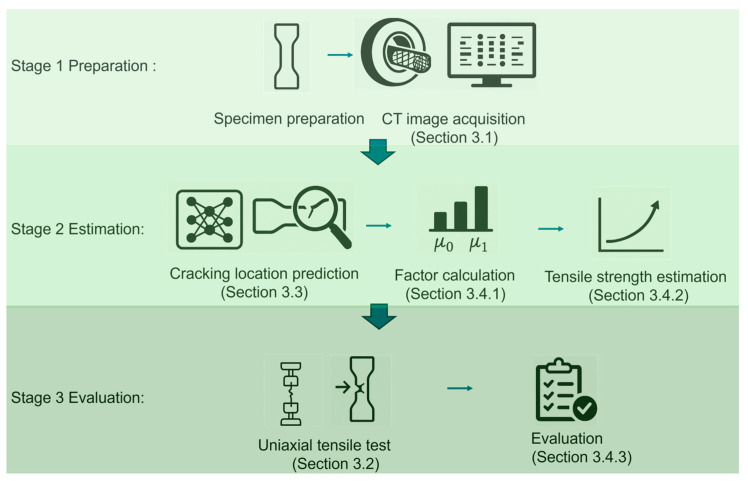
Overall research workflow of the proposed method.

An overview of the complete workflow is provided in Figure 1. The prepared UHPFRC dog-bone specimens are first subjected to X-ray computed tomography (CT) scanning, and the resulting CT images are processed to obtain fiber distribution data. A deep learning-based method is then used to predict the likely cracking location from these images. Based on the predicted location, the corresponding cross-section is analyzed to extract fiber orientation factors (*μ*_0_) and fiber efficiency factors (*μ*_1_), which are subsequently used to estimate the tensile strength of the specimen. To evaluate the approach, uniaxial tensile tests are performed to obtain the actual cracking location and measured tensile strength. The predicted and experimental results are compared to assess the accuracy of cracking location prediction and the reliability of strength estimation. This integrated approach enables the development of a reliable, non-destructive tensile strength estimation framework for UHPFRC based on predicted cracking locations.

### 2.1. Mixture Properties and Specimen Preparation

In this study, as shown in Table 2, the tested UHPFRC specimens were made from an industrial premix (J-THIFCOM, Osaka, Japan) consisting of a standard powder mix combined with a specialized mixing liquid. The mixture featured a 16% liquid-to-powder ratio, 2.5% by volume of steel wool, and 2.5% by volume of straight steel fibers, which measured 15.0 mm in length, 0.2 mm in diameter, and had a tensile strength of 2865 MPa. At 28 days, the UHPFRC exhibited an average elastic module of 39.5 GPa and a compressive strength of 151 MPa, measured on cylinders with a 50 mm diameter and 100 mm height.

**Figure 2:**
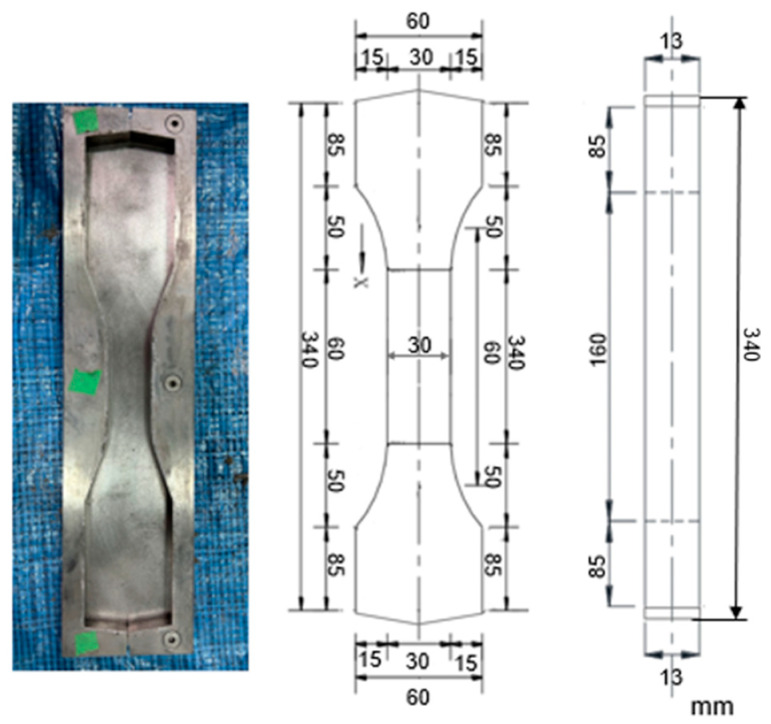
Mold and size of dog-bone specimen.

**Figure 3:**
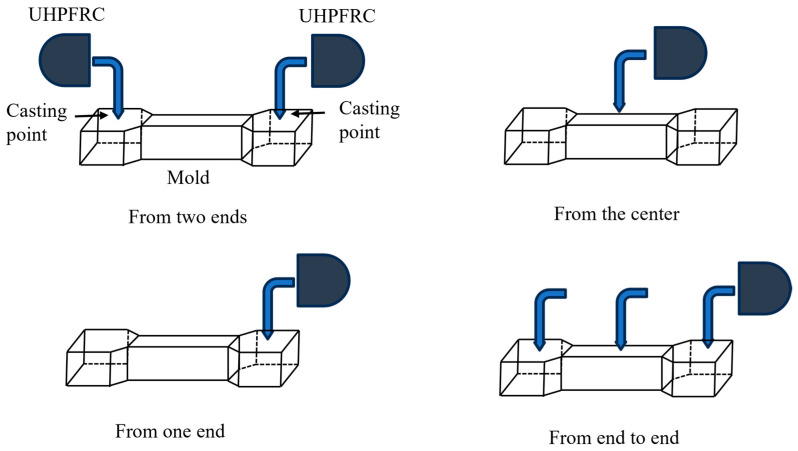
Four casting methods.

As shown in Figure 2, the fresh UHPFRC, which exhibited a slump flow of 258 mm, was cast into dog-bone-shaped molds with a total length of 340 mm. The gauge section measured 60 mm in length and maintained a constant cross-section of 30 mm × 13 mm, flanked by 50 mm transition zones. To achieve different fiber distribution characteristics, as shown in Figure 3 and Table 3, a total of 26 specimens were cast using four different casting methods. The number of specimens varied across casting methods according to the experimental objectives and the expected variability in fiber distribution. A larger number is assigned to U2E due to higher expected variability, a moderate number is assigned to UC given its near-isotropic fiber distribution, and fewer specimens are assigned to U1E and UEE based on existing knowledge of unidirectional fiber flow behavior and the anticipated minor differences between these casting types. After casting, the specimens were initially covered with plastic sheets to retain moisture and kept warm for 24 h. Subsequently, the molds were removed, and the specimens were subjected to moist curing at 20 °C and 100% relative humidity until testing. Specimens were tested at 81 days of age.

### 2.2. Uniaxial Tensile Test

#### 2.2.1. Test Method

**Figure 4:**
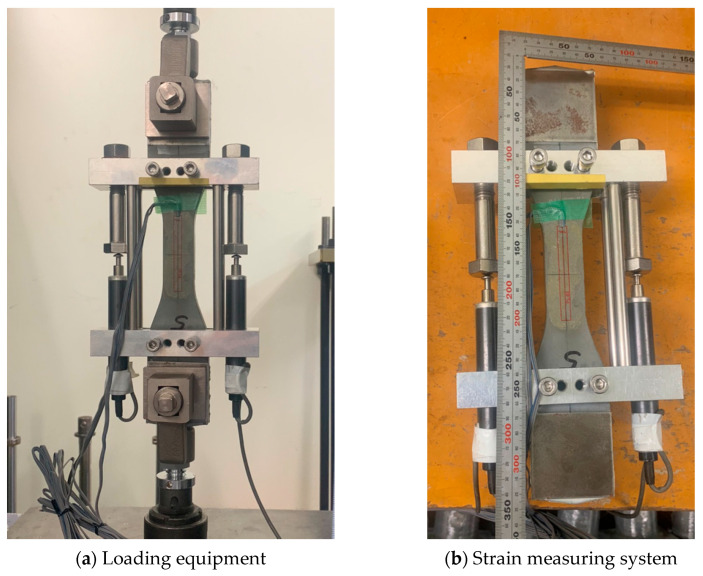
Test setup.

Before the uniaxial tensile test, all specimens were first affixed with 60 × 60 mm cast iron patches at both ends to prevent damage to the specimen ends by the fixture during testing and to avoid relative sliding caused by insufficient connection between the fixture and the contact surface of specimens. The uniaxial tensile test was conducted using a 50 kN electronic servo-hydraulic universal testing machine (Instron, Norwood, MA, USA) with displacement control, as shown in Figure 4a. The displacement rate was set to 0.05 mm/min before the peak stress and 0.5 mm/min after the peak stress. A specialized test frame was used to measure elongation on both sides of the specimen, employing two linear variable differential transformers (LVDTs) with a 150 mm gauge length. The average value of the two LVDTs was used to calculate the tensile strain, as illustrated in Figure 4b.

The preload applied a manually loaded tensile stress of 100 N to eliminate any potential gaps between the specimen and the fixture, ensuring full contact and proper alignment. Additionally, the position of the specimen within the testing device was stabilized to prevent any initial movement or misalignment during subsequent loading. The uniaxial tensile test was terminated when the residual stress dropped to 20% of the peak stress or when the displacement reached 30% of the fiber length.

#### 2.2.2. Measurement of Cracking Location

**Figure 5:**
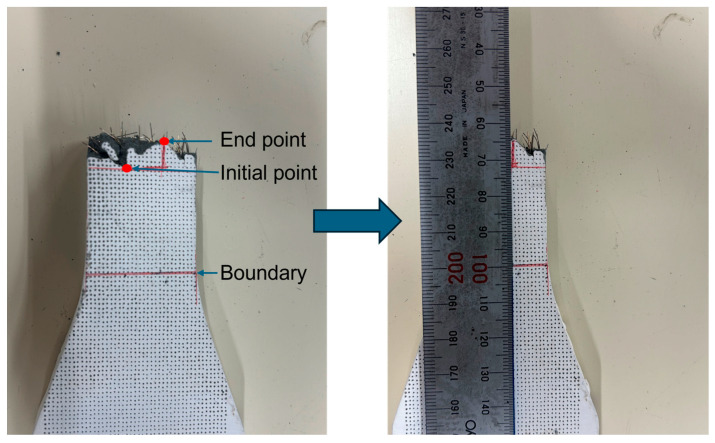
Measurement of cracking location.

After completing the UTT, the width of the cracking location for each specimen was measured. As illustrated in Figure 5, the procedure comprises the following steps: Initially, the boundary between the gauge length and the transition zone was identified, which serves as the starting point for the measurement. Then, based on this starting point, the crack initial point nearest to the boundary and the crack end point farthest from the boundary were determined. Finally, the perpendicular distance between the initial and end points was measured. This distance is defined as the width of the cracking location. This method ensures high repeatability and accuracy in the measurement of the width of the cracking location.

### 2.3. Fiber Characteristics Analysis

#### 2.3.1. X-Ray CT Scanning

**Figure 6:**
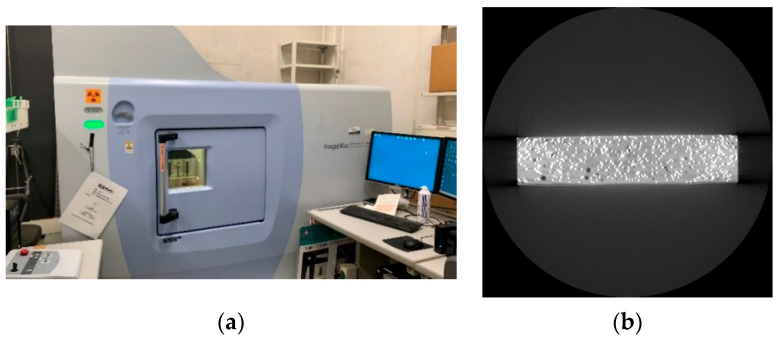
CT scan process. (**a**) Shimadzu inspeXio SMX-225CT FPD HR Plus scanner. (**b**) CT image of transversal section.

As shown in Figure 6, the specimens underwent X-ray scanning by SHIMADZU inspeXio SMX-225CT FPD HR Plus (Shimadzu Corporation, Kyoto, Japan), generating CT images encompassing fiber distribution, orientation, and count information. The fiber distribution was determined by the number of steel fiber pixels in each image, while the fiber orientation was assessed based on the area of each steel fiber pixel. Each scan produced 2644 CT images on the transversal section, each with an image resolution of 1024 × 1024 pixels.

#### 2.3.2. CT Images Analysis

**Figure 7:**
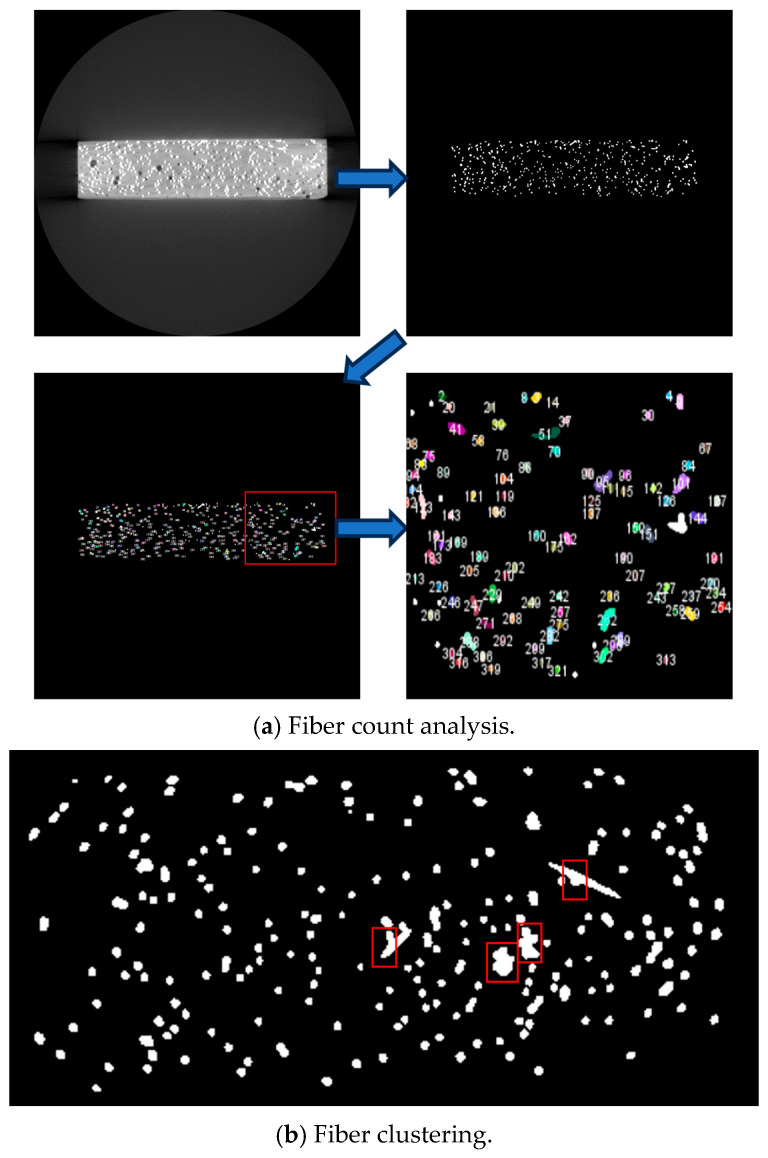
Image analysis.

The process of analyzing *μ*_0_ and $\bar{\mu_{1}}$ using the Fiji [34] image analysis software on CT scan images involves the following steps:

1. Binary conversion of CT scan images: As shown in Figure 7a, the CT scan images are first converted into binary images by adjusting the threshold to distinguish fibers from the background. This ensures that the fibers are clearly represented in the binary image.
2. Pixel-based fiber count: In the binary images, a known pixel-to-length scale was used (7 pixels = 0.2 mm). The white pixel count was measured to determine the number of fibers represented in each cross-sectional image.
3. Modification of CT image analysis results: As illustrated in Figure 7b, fiber clustering occasionally occurs during the casting process, as highlighted within the red boxes in the binarized CT image. This phenomenon introduces errors when analyzing fiber counts using image analysis techniques. To mitigate the impact of these errors on the results, manual visual inspection was employed to adjust the fiber count results obtained through image analysis software.
4. Calculation of *μ*_0_ and $\bar{\mu_{1}}$: Using the equation described in Section 2.4.2, the orientation factor *μ*_0_ is calculated for the image. Subsequently, based on the curve shown in Section 2.4.2, the corresponding efficiency factor ($\bar{\mu_{1}}$) is derived.

### 2.4. Cracking Location Prediction 

#### 2.4.1. Introduction and Setting of Deep Learning Technology

**Figure 8:**
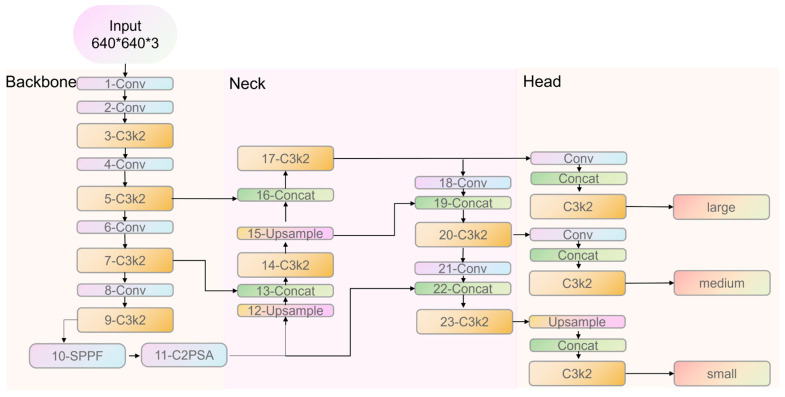
Framework of YOLOv11.

The deep learning model employed in this study is YOLOv11 (You Only Look Once, version 11), developed by Ultralytics [37,38]. YOLOv11 represents the latest advancement in the YOLO series for object detection, renowned for its efficiency and accuracy in real-time applications. This iteration improves upon its predecessors by enhancing object detection capabilities, offering superior performance and speed. YOLOv11 excels at identifying and classifying multiple objects within images through a single neural network pass, making it highly suitable for tasks requiring rapid and precise object detection. Its architecture incorporates Convolutional Layers (Conv), CSP Bottleneck with 2 convolutions (C3k2), Spatial Pyramid Pooling-Fast (SPPF), and CSP-PAN Spatial Attention (C2PSA), optimizing both detection speed and accuracy, and setting new benchmarks in computer vision. Figure 8 illustrates the YOLOv11 framework.

The codes were written in the Python 3.8 environment and implemented on an image-computing workstation equipped with a single NVIDIA GeForce RTX 3060Ti GPU (NVIDIA Corporation, Santa Clara, CA, USA), an Intel Core i7-12700 CPU @ 2.10 GHz (Intel Corporation, Santa Clara, CA, USA), and Windows 11. The hyperparameters were optimized based on Ultralytics’ YOLOv11 documentation [38], balancing GPU memory constraints and convergence stability. As shown in Table 4, these hyperparameters include a batch size of 4, a learning rate of 0.001 (reduced by a factor of 0.9 at the end of each iteration), a momentum of 0.937, a weight decay of 0.0005, a patience of 100, and a training epoch of 600. The training process employs the Adaptive Moment Estimation (Adam) optimizer, which combines momentum and RMSProp (Root Mean Square Propagation) techniques to ensure adaptive and efficient convergence.

#### 2.4.2. Cracking Location Prediction Method

The cracking location prediction process can be divided into four steps: (1) deep learning model training, (2) “defective distribution” detection, (3) detection result statistics, and (4) the visualization of prediction results.

**Figure 9:**
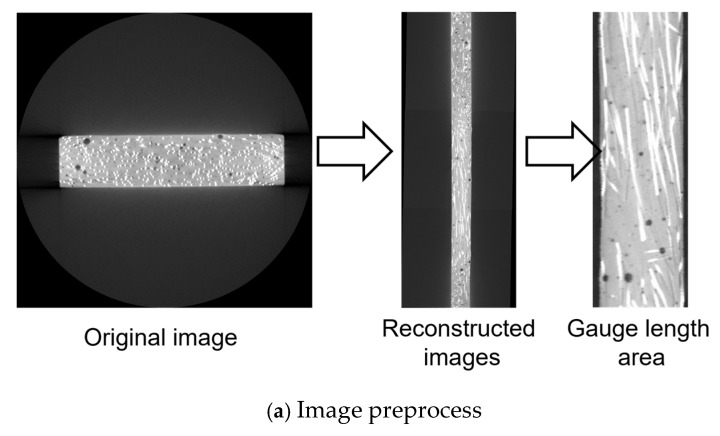

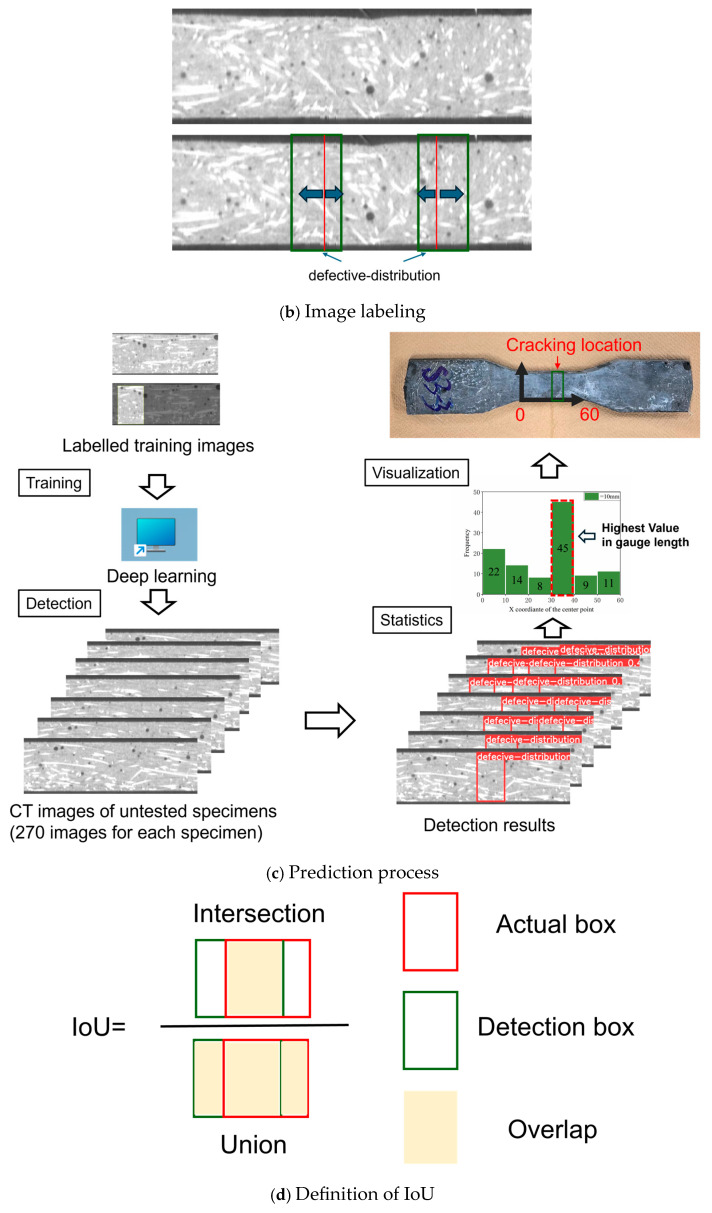
Cracking location prediction process.

Before using deep learning, CT images need to be preprocessed first. As shown in Figure 9a, the CT images of all specimens were reconstructed using Fiji to obtain longitudinal images. These images were then cropped to match the actual proportion of the specimen’s gauge length, and the excess black background was removed, retaining only the regions containing fiber information. Each specimen produced 270 images for use in the second step, each with a resolution of 467 × 185 pixels.

The image labeling process, as illustrated in Figure 9b, involved a thorough manual inspection of pre-processed CT images to identify “defective-distribution” regions. These regions were characterized by a significantly lower fiber count compared to surrounding areas or by the presence of large air bubble zones formed during the casting process (appearing as black voids in the CT images).

To determine the boundaries of label boxes, the central line of the area with a relatively lower fiber count was first visually identified (marked by the red line in the image). From this central line, the boundaries were extended laterally until the fiber pixel density exceeded 50% compared to the average fiber pixel density in the adjacent regions. Since the crack extends across the entire cross-section of the specimen, the height of the label box was defined as the specimen’s height. A total of 1350 images from five specimens (U2E-2, U2E-3, U2E-6, UC-2, and UEE-5) were randomly selected to form the dataset for deep learning and were labeled. Then, these images were subjected to data augmentation techniques, including rotation, flipping, cropping, and brightness adjustment [39], increasing the total dataset to 3750 images.

As shown in Figure 9c, the labeled images were first divided into training, validation, and test sets in a ratio of 7:2:1 for deep learning training. Then, the trained deep learning model was subsequently applied to detect “defective-distribution” on 270 images from each specimen outside the dataset, generating results with label information (the height, width, and the center point coordinates of the detection boxes.). After that, the detection results were statistically analyzed to produce a frequency distribution histogram of the X-coordinate of the center points of the detection boxes. Finally, the highest value in the histogram was selected as the predicted cracking location and visualized on the specimen. It should be noted that the coordinate system uses the gauge length as the origin of the abscissa.

For instance, as shown in the histogram of Figure 9c, the first bar has a y-value of 22 and an x-value range of 0–10. This indicates that in the region between x-values 0 and 10, 22 out of the 270 images were detected in this area as a “defective-distribution”.

In order to evaluate the results of cracking location prediction, refer to the definition of mean Average Precision (mAP) [40]. As shown in Figure 9d, the mAP@50 uses the 50% Intersection over Union (IoU) threshold to determine whether the detection box and the actual box match correctly. In this study, a prediction is deemed successful when the overlap rate between the detection box and the actual box reaches or exceeds 50%. It should be noted that the IoU will not be calculated if the crack in a specimen occurred entirely outside the gauge length. Conversely, if any portion of the crack extends beyond the gauge length, only the area within the gauge length will be considered for the actual box when calculating the IoU.

### 2.5. Tensile Strength Estimation

#### 2.5.1. Basic Formulation

For a strain-hardening UHPFRC, the estimation of tensile strength (*f_t_*) can be made by considering the fiber orientation factor (*μ*_0_) and efficiency factor (*μ*_1_) within the cracking location:

(7) $$f_{t}=\mu_{0}\mu_{1}\tau_{f}V_{f}\frac{l_{f}}{d_{f}}$$

Here, *τ_f_* denotes the interfacial bond strength that exists between the fibers and the surrounding cement matrix, *V_f_* represents the volume fraction of the fibers, and *l_f_*/*d_f_* represents the aspect ratio of the fibers [41,42].

#### 2.5.2. Fiber Orientation Factor

The orientation factor (*μ*_0_) is described as the probability of a single fiber to intersect a specific cross-sectional area. This factor is calculated using the actual fiber count that traverses a unit area (*n_f_*) compared to the expected count, as shown in Equation (8). In this equation, *A_f_* represents the fiber’s cross-sectional area:

(8) $$\mu_{0}=n_{f}\frac{A_{f}}{V_{f}}$$

From a stereological perspective, the value of *μ*_0_ equals 1.0 for fibers that are aligned unidirectionally along the tensile axis (1D), *μ*_0_ equals 2/π for fibers that are randomly oriented within a perfect plane (2D), and *μ*_0_ equals 0.5 for fibers randomly dispersed throughout a volume (3D).

#### 2.5.3. Fiber Efficiency Factor

The fiber efficiency factor (*μ*_1_) is characterized as the expected outcome of the fiber efficiency function, as shown in Equation (9). In this equation, *g(θ)* refers to the quotient of the pull-out force for a fiber inclined at an angle *θ* relative to the main stress direction, compared to a fiber that is perfectly aligned. Additionally, *f(θ)* denotes the probability density function for the orientation angle *θ* of the fibers intersecting the examined section:

(9) $$\mu_{1}=\int_{0}^{\frac{\pi }{2}}g\left(\theta \right)f\left(\theta \right)d\theta$$

**Figure 10:**
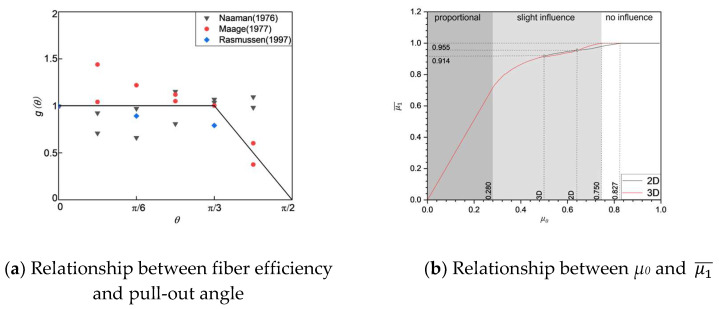
Fiber characteristics factors [41,42,43,44,45]. (**a**) Relationship between fiber efficiency and pull-out angle; (**b**) relationship between *μ*_0_ and $\bar{\mu_{1}}$.

A simplified model, proposed by C. Oesterlee [42], is depicted in Figure 10a: *g(θ)* = 1.0 for *θ* ≤ π/3; for angles greater than π/3 but less than or equal to π/2, *g(θ)* = −(6*θ*/π) + 3. The calculated *μ*_1_ based on [42], hereafter, is called average fiber efficiency factor $\bar{\mu_{1}}$. Bastien-Masse and colleagues [41] established a correlation between the average efficiency factor ($\bar{\mu_{1}}$) and the orientation factor (*μ*_0_) utilizing stereological principles, as demonstrated in Figure 10b. It is important to note that the impact of $\bar{\mu_{1}}$ on tensile strength diminishes progressively once *μ*_0_ exceeds 0.28; when *μ*_0_ reaches 0.75 or higher, this influence becomes negligible when $\bar{\mu_{1}}$ equals 1.0.

#### 2.5.4. Calculation of *μ*_0_ and $\bar{\mu_{1}}$

To determine the representative values of *μ*_0_ and *μ*_1_ for a given UHPFRC specimen or structural element, cracking surface image analysis of cut specimens has been widely adopted in the literature, despite being time-consuming [31,43,44,45,46,47]. In recent years, several non-destructive testing (NDT) methods [35,48,49,50,51,52,53,54], such as magnetic measurements, resistivity measurements, and AC impedance spectroscopy, have been developed for the rapid estimation of *μ*_0_ and *μ*_1_ in UHPFRC laboratory specimens and structural elements. More recently, hybrid electromagnetic and imaging-based NDT approaches have emerged 47,51], offering improved resolution and robustness in evaluating fiber orientation and distribution in structural-scale UHPFRC elements. These NDT methods offer significant potential for improving testing efficiency and enabling quality control. However, NDT methods typically rely on indirect data acquisition, leading to lower accuracy compared to traditional destructive methods. Additionally, their results are often affected by environmental conditions (e.g., humidity and temperature) and material properties (e.g., fiber type and volume fraction). Moreover, the high cost and complexity of some NDT devices limit their broader application. In this study, a deep learning-based method is employed to predict cracking locations followed by image analysis techniques to evaluate *μ*_0_ and $\bar{\mu_{1}}$ at the predicted cracking locations. This method addresses the accuracy limitations of NDT methods while improving data acquisition efficiency.

## 3. Results and Discussion

### 3.1. Modification Results of CT Image Analysis

**Figure 11:**
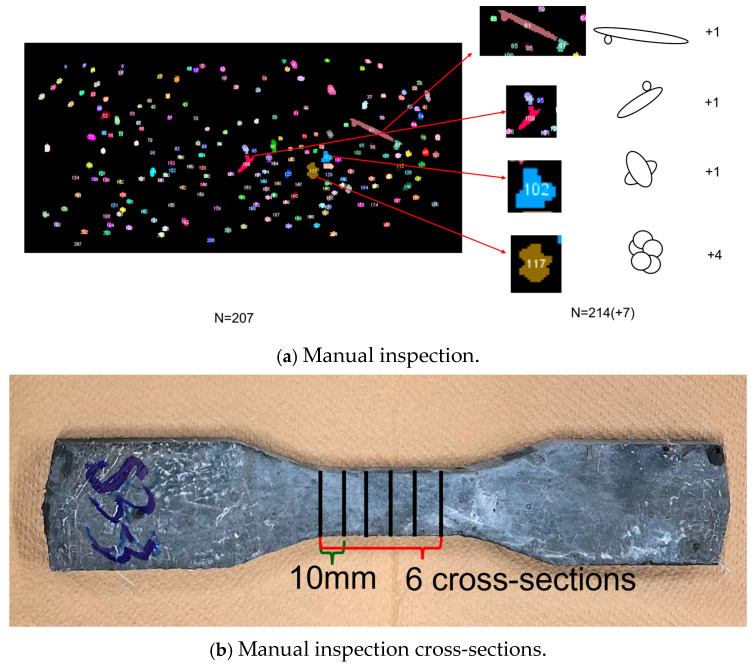
Modification example.

As shown in Figure 11a, the cross-section at the 50th mm mark of specimen U1E-1 initially yielded a fiber count of *N* = 207 through image analysis software. However, upon manual visual inspection, it was evident that some clustered fibers had been mistakenly identified as individual ones. The fiber count was subsequently adjusted to *N =* 214 (+7).

It is important to note that, due to the large number of CT images within the gauge length area (800 images per 60 mm), manually inspecting each image would be time-consuming and labor-intensive. To address this, as shown in Figure 11b, one cross-section every 10 mm was selected for manual inspection, resulting in six cross-sections per specimen. For each of the four casting methods, three specimens were selected, totaling 72 cross-sections (72 CT images) for manual inspection across 12 specimens. The final adjusted fiber counts for each casting method were calculated by averaging the results of these inspections. The results are presented in Table 5.

### 3.2. Uniaxial Tensile Test (UTT) Results

**Figure 12:**
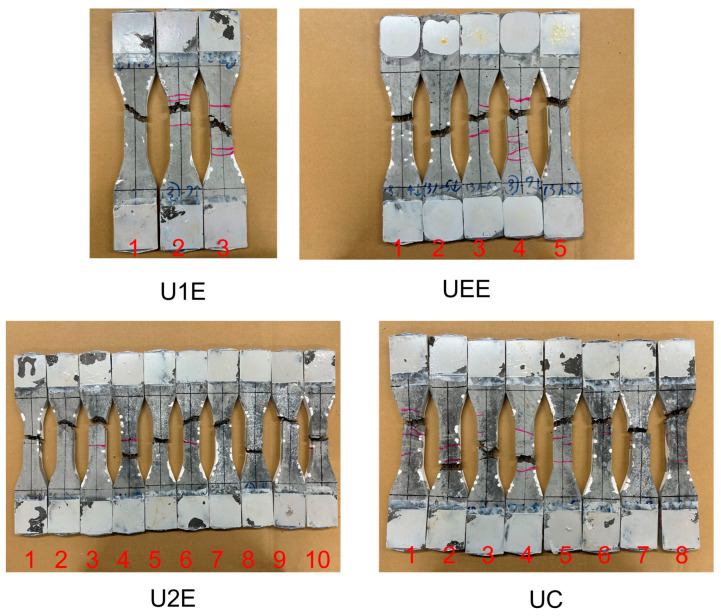
Specimens after UTTs.

**Figure 13:**
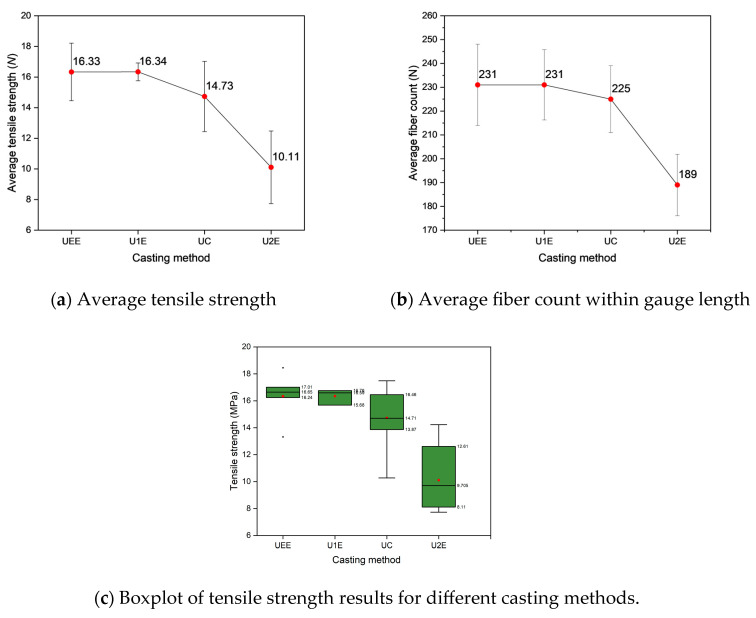
Influence of different casting methods.

**Figure 14:**
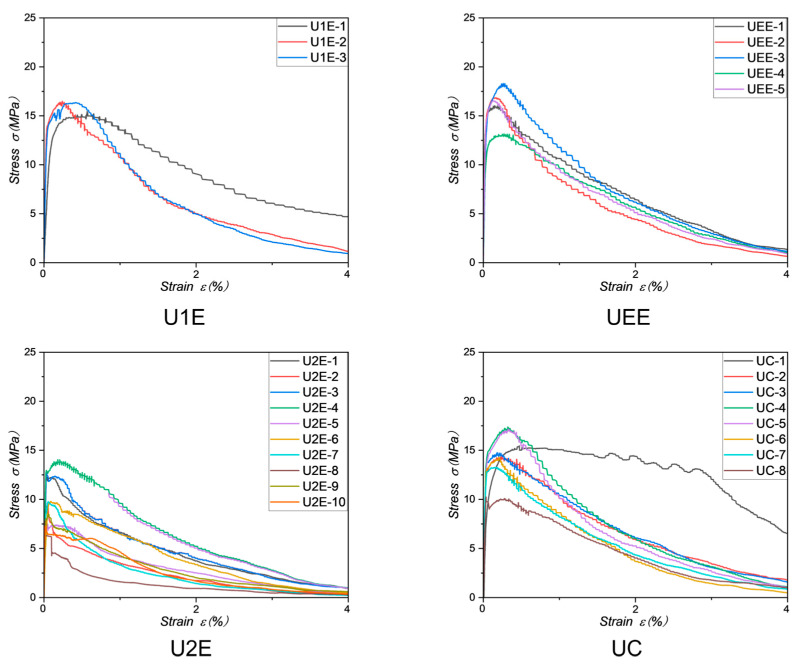
Stress–strain response of four groups.

**Figure 15:**
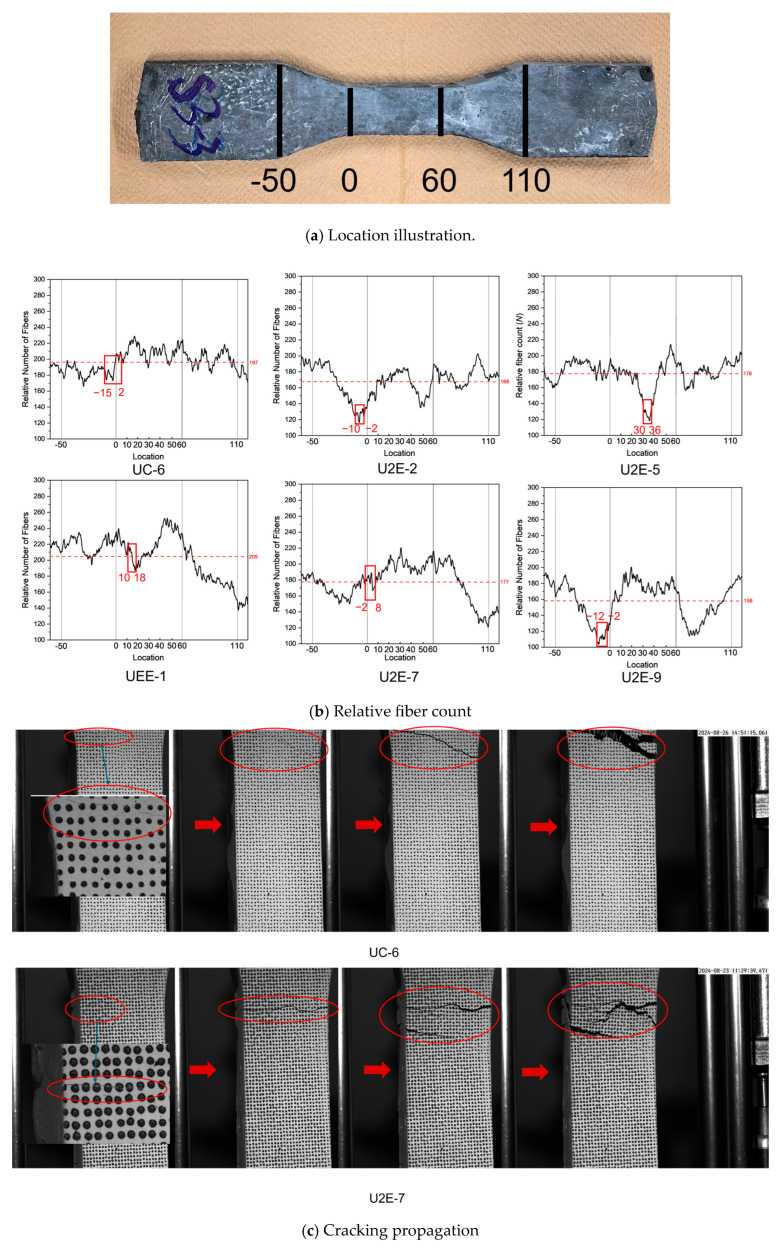
Fracture behavior analysis.

The results of the uniaxial tensile tests are summarized in Figure 12, Figure 13, Figure 14 and Figure 15 and Table 6. The influence of fiber distribution on tensile strength and fracture behavior was systematically investigated based on specimen appearance, average tensile strength, fiber count, stress–strain responses, and fiber distribution along the specimen length.

#### 3.2.1. Relationship Between Tensile Strength and Fiber Count

As shown in Table 6, and Figure 12 and Figure 13, the tensile strengths of UHPFRC specimens under different casting methods were analyzed. Figure 12 shows the post-test appearances of the specimens. Specimens from the U1E and UEE groups exhibited multiple fine cracks distributed within the gauge length, indicating strain-hardening behavior. In contrast, most U2E specimens displayed wider cracks, often occurring outside the gauge length, suggesting strain-softening failure. These visual observations underline the influence of fiber distribution on crack patterns and overall mechanical response.

Figure 13a illustrates the average tensile strength for each specimen group, while Figure 13b depicts the average fiber count per cross-section within the gauge length for each specimen group.

A strong correlation was observed between average tensile strength and fiber count within the gauge length. The specimens cast using the UEE and U1E methods achieved the highest tensile strengths, 16.33 MPa and 16.34 MPa, respectively, with an average fiber count of 231 fibers per cross-section. In contrast, U2E specimens exhibited the lowest tensile strength (10.01 MPa) and the lowest fiber count (189 fibers per cross-section). Specimens cast using the UC method demonstrated intermediate performance, with a tensile strength of 14.73 MPa and an average fiber count of 225 fibers per cross-section.

Figure 13c presents boxplots of tensile strength results by casting method. Specifically, specimens cast using the UEE and U1E techniques exhibited higher median tensile strengths and narrower interquartile ranges, indicating more consistent mechanical behavior. Conversely, specimens from the UC and U2E groups displayed wider distributions and greater variability—with the U2E group showing the lowest median tensile strength and the largest scatter—thereby reflecting the influence of non-uniform fiber distribution.

These results underscore the significant influence of casting methods on tensile performance, primarily by affecting fiber count within the gauge length. Specimens with a higher fiber count, as seen in the UEE and U1E methods, exhibited superior tensile performance, while the U2E method, with its lower fiber count, resulted in reduced tensile strength.

#### 3.2.2. Relationship Between Fiber Count and Fracture Behavior

In the investigation of fracture behaviors under tensile testing for UHPFRC, as shown in Figure 14, the groups U1E, UEE, and UC predominantly exhibited strain-hardening fracture modes, with UC-8 being the sole exception. Conversely, the U2E group, with the exception of U2E-1 and U2E-4, predominantly displayed strain-softening fracture modes.

Figure 14 presents the stress–strain responses for the different groups. Strain-hardening specimens typically show a significant post-peak deformation, maintaining stress after the first cracking, while strain-softening specimens exhibit a sharp stress drop immediately after peak stress. This behavior reflects the underlying fiber distribution and its effectiveness in bridging cracks under tensile loading.

As shown in Figure 15a, an in-depth analysis was conducted on the fiber count across each cross-section, spanning from one transition zone to the other (−50 mm to 110 mm). To facilitate a more effective comparison of fiber counts across different areas, the transition zones were approximated as trapezoids with top and bottom bases of 30 mm and 60 mm, resulting in a uniform linear transition in cross-sectional area. By normalizing the cross-sectional area within the gauge length to be 1, the relative area at the specimen ends was calculated as 2. This approach enabled the creation of a diagram illustrating the distribution of relative fiber counts across the specimens.

Figure 15b presents the relative fiber count distribution for representative specimens, with the actual cracking locations indicated by red boxes and annotated with red subscripts. Among the multiple minimum points observed in the Relative Number of Fibers–Location curve, the point highlighted with the red square was selected because it occurred within or immediately adjacent to the gauge length. This local minimum corresponds to the location where the final visible crack was ultimately formed. Since the objective of this study is to analyze and predict the final cracking location of UHPFRC specimens, only the local minimum directly associated with this critical cracking location was considered for analysis. Other local minima, which may correspond to minor cracking events outside the gauge length, were not selected as they are not representative of the dominant fracture behavior.

Specimens characterized by strain-hardening behavior (UEE-1 and UC-6) generally exhibited a higher relative fiber count (205 and 197), indicating a concentration of fibers in the gauge length and transition areas, and reduced content at the ends. This distribution allows for better utilization of fibers under tensile stress, resulting in strain-hardening fracture modes.

In contrast, specimens displaying strain-softening behavior (U2E-2, U2E-5, U2E-7, and U2E-9) had a notably lower relative fiber count (168, 178, 177, and 158, respectively), with apparent defect areas. For instance, near 35 mm in U2E-5, a significant drop in fiber count was observed, facilitating crack formation. Additionally, these specimens exhibited fewer fibers distributed in the transition and gauge length areas, with a substantial concentration of fibers at the specimen ends. This uneven distribution led to inadequate fiber bridging under tensile stress, resulting in strain-softening fracture modes.

Figure 15c further shows representative cracking propagation paths. In specimens like UC-6 and U2E-9, cracks initiated at regions of lower fiber concentration, particularly in transition zones, and then propagated into the gauge length. The initiation of cracks in fiber-deficient zones highlights the importance of maintaining a sufficient fiber distribution not only within the gauge section but also near transition areas to prevent premature failure.

Furthermore, for specimens in which cracking occurred outside the gauge length (U2E-2 and U2E-9, as illustrated in Figure 15b), a significant reduction in fiber count was observed in the transition zone compared to the gauge length region. This reduction in fiber count created a defect zone within the transition region, which ultimately served as the nucleation site for crack formation. For specimens in which cracking occurred from the transition zone to the gauge length (including U1E-2, U2E-4, U2E-7, UC-1, UC-5, and UC-6), a detailed analysis of their cracking propagation patterns was performed to investigate both the underlying mechanisms and causes of cracking propagation. Comparison of the cracking propagation images (as shown in Figure 15c) revealed that the cracking propagation behavior of transition zone-dominated specimens, such as U1E-2, U2E-4, UC-1, UC-5, and UC-6 (with UC-6 serving as a representative example), is similar to that observed in specimens U2E-2 and U2E-9, where cracking occurs outside the gauge length. This phenomenon arises because the fiber count in the transition zone of these specimens is significantly lower than that in the adjacent gauge length region, thereby forming a defect zone. Consequently, the defect zone initiates cracking in the transition zone, which subsequently propagates into the gauge length region during testing. For specimen U2E-7, however, the crack initially appeared in a region within the gauge length where the fiber count was significantly lower than in other areas. Subsequently, the crack propagated gradually from the gauge length region toward the transition zone.

In contrast, specimens U2E-5 and UEE-1 did not exhibit any significant decrease in fiber count near the transition zone adjacent to the gauge length, in comparison to the gauge length region. Consistent observations across other specimens indicate that the locations of crack initiation are closely correlated with the fiber count.

Specifically, at the cracking sites, the relative fiber count typically decreases gradually or exhibits a pronounced drop compared to the surrounding areas. This phenomenon occurs because cracks tend to initiate in regions with fewer fibers, where the tensile strength is reduced and the matrix bears most of the tensile stress. Consequently, the local stress more easily reaches the concrete matrix’s ultimate tensile strength, leading to early cracking. Furthermore, due to the weakened fiber bridging effect, the cracks are less effectively constrained, increasing their possibility of forming and propagating. Similar findings have been reported in the literature, including studies by Shen et al. [33], reinforcing the critical relationship between fiber count and crack development in UHPFRC.

### 3.3. Cracking Location Prediction Results

#### 3.3.1. Deep Learning Model Performance

Evaluating the performance of a YOLO object detection model for a single-class task involves several key metrics that reflect the accuracy and reliability of the model. The primary evaluation metrics include mean Average Precision (mAP), recall, and the F1 score [41]. For a single-class detection task, mAP serves as a critical metric for assessing precision. Specifically, mAP@0.5 measures precision at an Intersection over Union (IoU) threshold of 0.5, evaluating how well the predicted bounding boxes align with the ground truth under moderate overlap conditions. In contrast, mAP@0.5:0.95 provides a stricter and more comprehensive assessment by averaging precision across multiple IoU thresholds, ranging from 0.5 to 0.95 in steps of 0.05.

Recall measures the proportion of ground truth objects that are successfully detected, ensuring that the model minimizes missed detections. The F1 score, calculated as the harmonic means of precision and recall, provides a balanced measure of the model’s performance by accounting for both false positives and false negatives.

To evaluate the performance of the YOLOv11 model on the “defective distribution” detection task, experiments were conducted using the proposed dataset. Key metrics, including mAP, recall, and the F1 score, were utilized to comprehensively assess the model’s accuracy and reliability. The results of these evaluations are summarized as follows:

The YOLOv11 model achieved a mAP@0.5 of 0.87. When considering the stricter metric of mAP@0.5:0.95, which has average precision over IoU thresholds ranging from 0.5 to 0.95, the model attained 0.52. These results demonstrate the model’s capability to detect the target object with high accuracy across a range of IoU thresholds.

**Figure 16:**
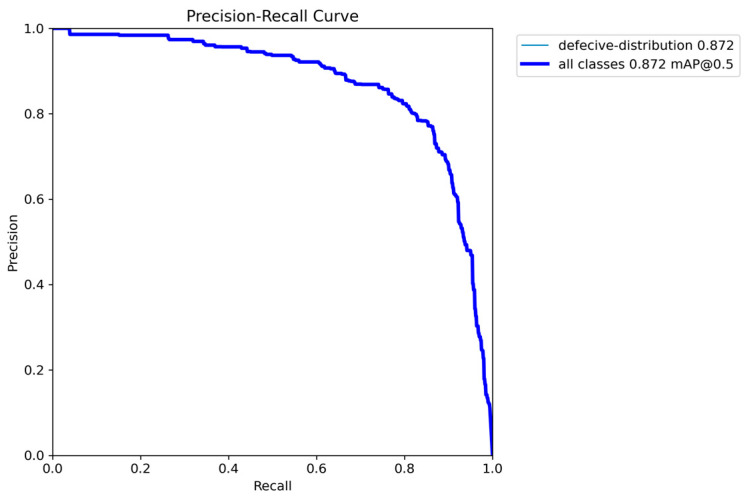
P-R curve of YOLOv11 model.

In addition to precision metrics, the model achieved a recall of 0.98, reflecting its ability to detect a large proportion of ground truth objects. The balance between precision and recall was confirmed by an F1 score of 0.81, indicating consistent performance in minimizing false positives and false negatives. Precision–Recall (PR) curves, shown in Figure 16, visualize the model’s ability to maintain high precision and recall across different confidence thresholds.

Although the overall performance is promising, there is room for further improvement. Enhancements such as integrated data augmentation strategies or advanced post-processing techniques could be explored to achieve better model performance.

#### 3.3.2. Cracking Location Prediction Results and Evaluation

**Figure 17:**
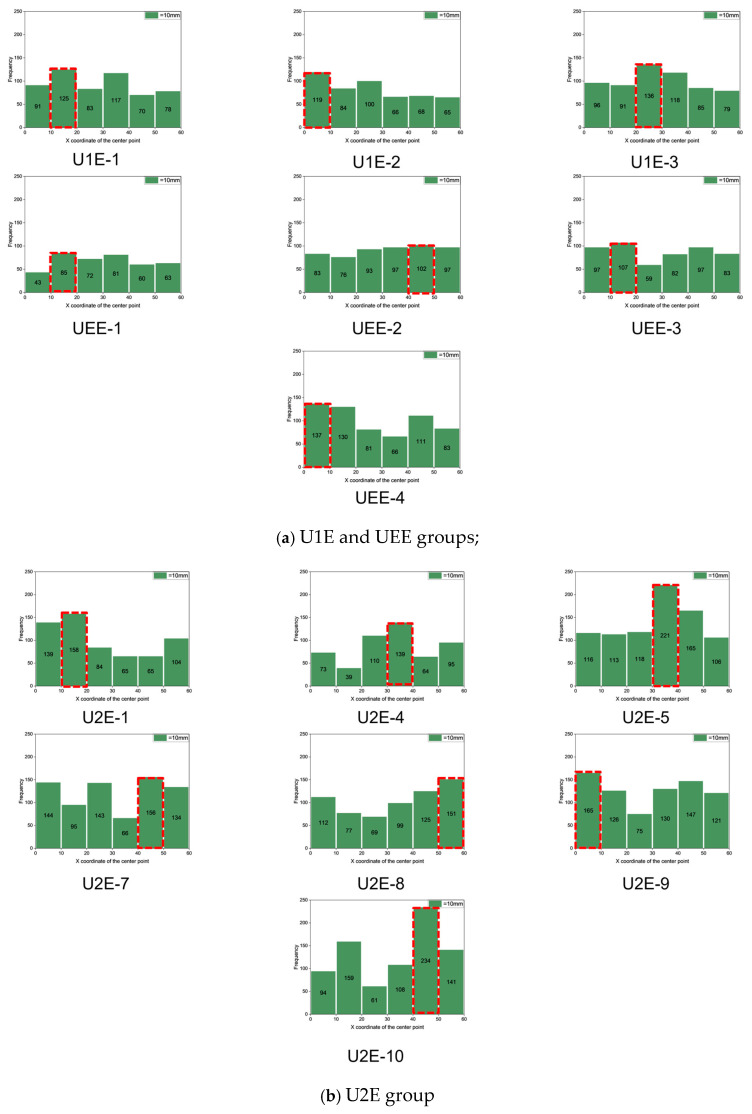

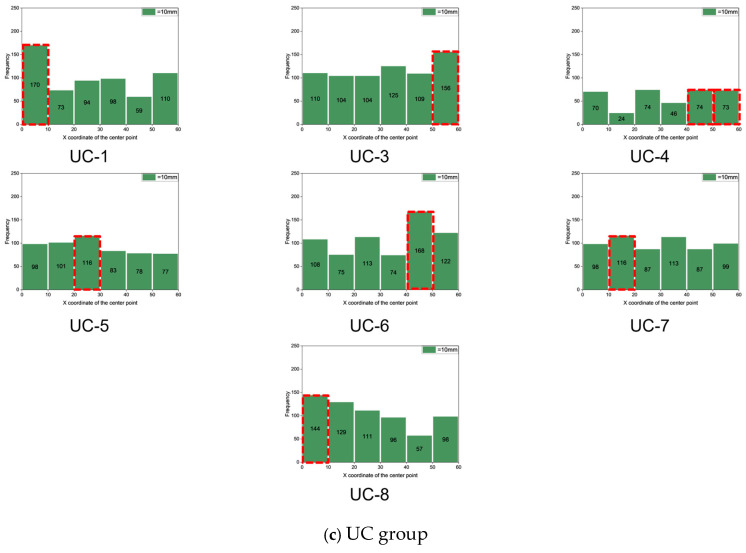
Statistical histograms of detection results.

Through the histograms shown in Figure 17, the cracking location prediction results presented in Table 7 were obtained. Combined with the failure modes of the specimens and Table 7, the following observations were made:

As shown in Figure 17, statistical histograms differ across casting methods. For the U1E and U2E groups, the histograms exhibit relatively distinct peaks. In contrast, the distributions for the UEE and UC groups appear to be comparatively flatter, with several bins showing similar frequencies. In this study, the bin with the highest count was selected to represent the predicted cracking location. While this selection strategy is appropriate when the peak is pronounced, it may introduce uncertainty for flatter distributions. To maintain methodological consistency across all specimen groups, the same selection approach was applied throughout the analysis. However, it is acknowledged that more robust criteria—such as threshold-based or clustering-based selection—should be explored in future work to enhance the reliability of cracking location prediction under low-gradient distributions.

Specimens cast using the UEE and U1E methods demonstrated a prediction success rate of 100%, with all seven specimens successfully predicted and the majority achieving an overlap rate above 50%. In the UEE group, all specimens were successfully predicted, with UEE-1 and UEE-3 each achieving 60% and 80% overlap rates. Similarly, U1E-3 achieved a 67% overlap rate, while U1E-2 and UEE-4 met the success threshold with 50%. The strain-hardening behavior observed in these specimens confined cracks within the gauge length, enabling the proposed cracking location prediction method to effectively predict cracking locations. These results confirm the robustness of the model in predicting cracking locations for strain-hardening UHPFRC, aligning well with experimental observations.

**Figure 18:**
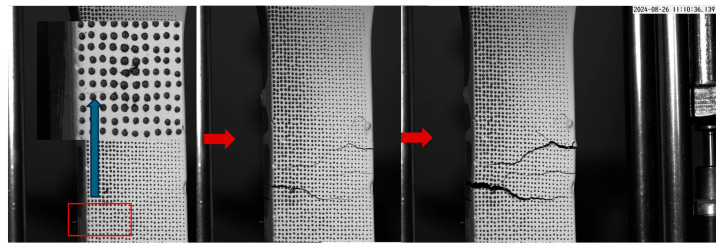
Cracking propagation process of UC-3.

The UC group exhibited 50% prediction success, with three out of six specimens meeting the success criterion. Accurate predictions, such as those of UC-1, UC-4, and UC-7, were primarily associated with specimens exhibiting strain-hardening behavior. For cases where predictions were unsuccessful, consider UC-3 as an example. The statistical graph of UC-3 reveals that the bar representing the highest value is not significantly different from the other bars, and an additional distinct peak is observed in the 30–40 mm interval. As shown in Figure 18, the cracking propagation process of UC-3 was observed. This observation suggests that multiple defective regions exist within the observed range, and these regions eventually coalesce to form an extensive crack spanning from 30 mm to 55 mm. Given that the predicted crack width in this study was fixed at 10 mm, the overlap rate for UC-3 is only 17%. Similarly, for UC-5, UC-6, and the unsuccessfully predicted UC-8, as described in Section 3.2.2, crack formation was primarily attributed to a significant reduction in fiber count in the transition region adjacent to the gauge length compared to the fiber count in the gauge length region. This led to the initial crack formation in the transition zone, which subsequently extended into the gauge length region. This phenomenon occurs because cracks typically initiate in areas with fewer fibers, where the tensile strength is lower and the tensile stress is primarily borne by the matrix. As a result, the local stress more easily reaches the ultimate tensile strength of the concrete matrix, leading to early cracking. Meanwhile, due to the weakened fiber bridging effect, cracks are less effectively constrained, making them more likely to form and propagate. Consequently, the crack formation was not due to failure within the gauge length region, explaining the significant discrepancy between the actual crack location and the predicted location in this study.

**Figure 19:**
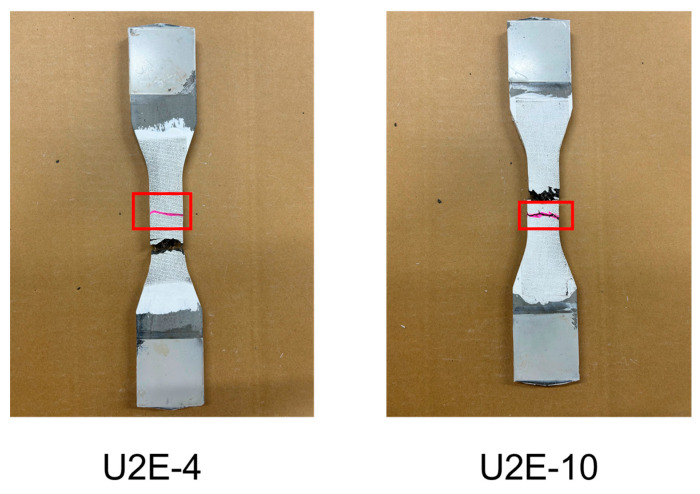
The cracks of U2E-4 and U2E-10.

In contrast, the U2E group displayed the lowest success prediction, with only two out of seven specimens successfully predicted, achieving a success rate of 28.6%. Only U2E-1 and U2E-5 achieved overlap rates above 50%, while most failed to meet the success criteria or exhibited cracks outside the gauge length. Based on the observations illustrated in Figure 19, the UTT results for specimens U2E-4 and U2E-10 revealed that, in addition to the primary crack, other cracks (within the red boxes) were present within the predicted cracking locations of 30–40 mm and 40–50 mm, respectively. As described in Section 3.2.2, the crack in specimen U2E-4 was observed to initiate in the transition zone and gradually extend into the gauge length region. Importantly, the ultimate crack formation in the gauge length region was not a direct consequence of an inherent weakness in that area; instead, it emerged from the propagation process initiated by the initial crack in the transition zone.

**Figure 20:**
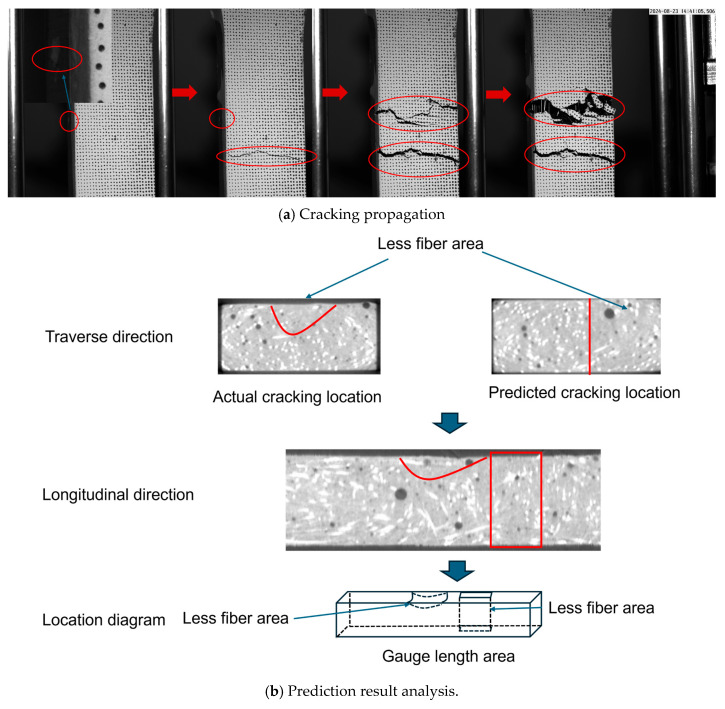
Failed prediction analysis of U2E-10.

As shown in the CT image of the actual cracking location in Figure 20, the fiber distribution in the 28–36 mm region (the actual cracking location) of U2E-10 exhibited a distinct pattern: the upper portion had fewer fibers, whereas the lower portion showed a higher fiber density. During the tensile test, the sparse fiber presence in the upper portion caused the matrix to reach its tensile limit earlier, leading to the formation of the initial crack. Subsequently, the crack propagated into the fiber-rich region, where the fibers began to exert a bridging effect that restricted further crack growth. Consequently, a new crack initiated in the relatively fiber-deficient lower region (40–50 mm). In the later stages of the test, the fibers in the 40–50 mm region developed a stronger bridging effect, effectively inhibiting further crack propagation. Meanwhile, in the 28–36 mm region, the increasing stress prevented the fibers from effectively halting crack growth, ultimately leading to the final crack formation in that region.

The failure in prediction, as illustrated in Figure 20b, can be attributed to the fiber distribution at the actual cracking location. Specifically, the upper part of this region (inside the red curve) contained relatively fewer fibers, whereas the bottom part exhibited a higher fiber density. Conversely, at the predicted cracking location, the fiber distribution on the right side (i.e., to the right of the red line) was relatively sparse. When the image was reoriented from the transverse direction to the longitudinal direction, the following phenomenon was observed: at the actual cracking location, the upper region contained fewer fibers while the lower region contained more; in contrast, at the predicted cracking location, a defective distribution was observed, spanning a height equivalent to the specimen’s total height. This discrepancy resulted in an increased number of detection outputs at the predicted cracking location during the deep learning-based detection process, ultimately causing a prediction failure. The primary cause of this failure was that, in the deep learning dataset, the labeled box height was set equal to the specimen’s height, which led to erroneous detection results. This issue underscores the dataset’s limitations in accurately representing real-world conditions. Future work will focus on refining the dataset to improve prediction accuracy.

In contrast, for specimen U2E-7, the differences among the data bars in Figure 17 were not sufficiently pronounced, which led to the prediction failure. In most U2E specimens, the absence of strain-hardening behavior (five out of seven specimens) contributed to diffuse and irregular crack propagation, thereby undermining the predictive method’s accuracy and reliability.

**Figure 21:**
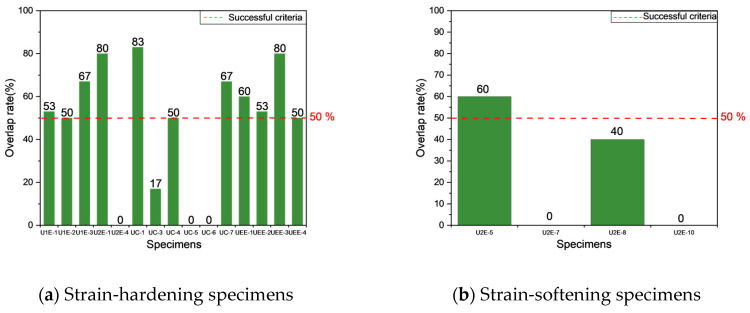
The overlap rates of different fracture behaviors.

As shown in Figure 21, the cracking location overlap rate of specimens with different fracture behaviors were analyzed. For specimens exhibiting strain-hardening behavior, eleven out of fifteen specimens achieved an overlap rate above 50%. This resulted in a prediction success rate of 73%. However, for strain-softening specimens, only one out of six reached or exceeded an overlap rate of 50%. Among the others, one specimen had an overlap rate of 40%, which does not meet the 50% criteria; two specimens had an overlap rate of 0%, and another two experienced cracks outside the gauge length. Consequently, this resulted in an overall prediction success rate of 17%.

The proposed cracking location prediction method demonstrated strong capability in predicting cracking locations for strain-hardening UHPFRC. In specimens exhibiting strain-hardening behavior, such as those from the UEE and U1E groups, the model consistently aligned predicted cracking locations with experimental results, achieving a prediction success rate of 73%. The ability to confine cracking locations within the gauge length ensured high prediction success rates. For strain-softening specimens, such as those in the U2E group, the performance of the model was limited due to the diffuse and irregular crack propagation. These findings emphasize the suitability of the proposed method for predicting cracking locations in strain-hardening UHPFRC, while underscoring the need for additional considerations in cases involving strain-softening behavior.

### 3.4. Tensile Strength Estimation Results

Typically, the cracking location of a UHPFRC specimen spans a defined width rather than occurring across a single cross-sectional area. This observation aligns with the findings in Section 3.2, which indicate that the fiber count within a specific region significantly influences the cracking location of the specimen.

To account for this behavior, subsequent tensile strength estimations in this study utilize an approach that averages the fiber count across all cross-sections within both the predicted and actual cracking locations. By averaging fiber counts across the cracking location, this method provides a more accurate representation of the structural interactions and stress distributions that contribute to the ultimate failure of the material.

#### 3.4.1. Interfacial Bond Strength Calculation

Due to experimental limitations, directly measuring interfacial bond strength (*τ*_*f*_) between fibers and the cementitious matrix in UHPFRC specimens was not feasible. Instead, *τ*_*f*_ was estimated indirectly using the experimentally determined tensile strength *f_t_* and key fiber parameters derived from CT image analysis. By rearranging Equation (7), *τ*_*f*_ was calculated as follows:

(10) $$\tau_{f}=\frac{f_{t}}{\mu_{0}\mu_{1}V_{f}\frac{l_{f}}{d_{f}}}$$

Fiber orientation factor (*μ*_0_) and efficiency factor (*μ*_1_) were derived from the CT image analysis of the predicted cracking areas.

As shown in Table 8, data from three randomly selected specimens (UC-3, U2E-1, and UEE-3) were used for *τ*_*f*_ calculation, yielding an average *τ*_*f*_ value of 12.50 MPa (slightly larger than 12 MPa [5] (fiber length/diameter: 13 mm/0.2 mm)).

In the next step, this value will be used to estimate the theoretical tensile strength (*f_ta_*) at the actual crack location and the estimated tensile strength (*f_te_*) at the predicted crack location. These estimated values will then be compared with experimental tensile strength (*f_t_*).

#### 3.4.2. Error Analysis of the Theoretical Tensile Strength

Equation (7) assumes a uniform distribution of fibers within the material. However, in actual processing and manufacturing, factors such as fiber flowability, interfacial interactions, and the geometric constraints of the structural component can induce local variations in fiber orientation and volume fraction. These deviations may affect the accuracy of tensile strength estimations, thereby leading to errors between the estimated tensile strength (*f_te_*) and experimental tensile strength (*f_t_*).

To address this issue, this study first calculates the theoretical tensile strength (*f_ta_*) at the actual cracking locations, which serves as a baseline reference. In actual cracking locations, fibers undergo realignment under external loading, ensuring that the orientation and volume fraction more accurately reflect the material’s behavior under applied stress. Therefore, the tensile strength in this region provides a more reliable representation of the fiber reinforcement effect and serves as a benchmark for the estimated tensile strength (*f_te_*).

Table 9 compared the theoretical tensile strength (*f_ta_*) with the experimental tensile strength (*f_t_*) in order to quantify the influence of fiber distribution on tensile strength. This approach effectively compensates for the errors introduced by the uniform distribution assumption, ensuring that the estimated values align more closely with the actual mechanical behavior of the material, thereby improving the accuracy and engineering applicability of the computational model.

**Figure 22:**
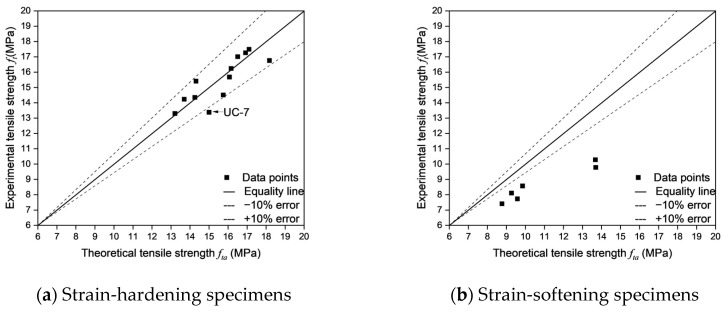
Error comparisons of different fracture behaviors.

As shown in Figure 22, the errors between the theoretical tensile strength and the experimental tensile strength were calculated for different fracture modes. Except for UC-7, the calculation errors for all strain-hardening specimens are within 10%, which falls within the acceptable range. This indicates that Equation (7) can accurately estimate the tensile strength of strain-hardening specimens.

**Figure 23:**
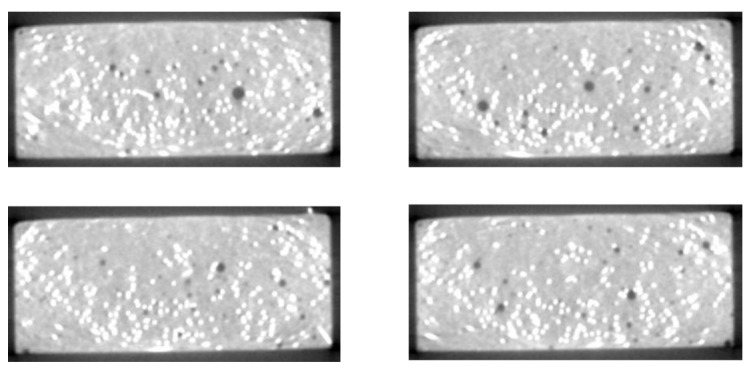
Fiber distribution at actual cracking locations of UC-7.

For UC-7, as shown in Figure 23, the CT images of the actual cracking locations reveal a notably poor fiber distribution in this region, with some areas at the top of the specimen completely devoid of fibers. This observation stands in stark contrast with the tensile strength formulas Equation (7), which typically assume a uniform fiber distribution—i.e., that fibers across all regions contribute equally to load bearing and crack bridging. In practice, however, fiber distribution is frequently heterogeneous; for example, CT images indicate that the upper region of the specimen is sparsely reinforced, whereas the lower region exhibits a denser fiber arrangement. Consequently, the fiber-sparse areas demonstrate inadequate bridging capability, resulting in elevated localized stresses under tensile loading, given that the matrix’s tensile strength is substantially lower than that of the fibers. As a crack initiates in such a vulnerable area, the insufficient fiber reinforcement cannot effectively impede further crack propagation, leading to significant stress concentration and premature local failure. This discrepancy between the assumed uniform stress distribution in the formulas in the literature and the actual localized failures and stress concentrations ultimately leads to estimated tensile strengths that exceed those measured experimentally.

In contrast, for strain-softening specimens, the theoretical tensile strengths are consistently higher than the experimental tensile strengths and the errors are larger than 10%. This overestimation arises from Equation (7)’s inherent assumption of strain-hardening behavior, which overemphasizes fiber contributions. In strain-softening specimens, once the initial crack forms, the material fails to sustain its stress level, and its load-bearing capacity gradually declines as the crack propagates. This behavior contrasts with that of strain-hardening specimens, which, after crack formation, continue to bear higher tensile loads through fiber bridging and demonstrate greater toughness during crack propagation. The inherent nature of strain-softening specimens implies that once a crack forms, the matrix’s contribution rapidly diminishes, rendering the specimen’s load-bearing capacity almost entirely dependent on fiber bridging. However, in actual tests, the fiber bridging effect often falls short of the idealized state assumed in computational models, resulting in experimentally measured tensile strengths that are lower than theoretical predictions.

This overestimation primarily arises from several key assumptions in the estimations. Firstly, the model assumes that all fibers are uniformly distributed and fully contribute to the bridging effect. However, in strain-softening specimens, rapid crack propagation induces localized stress concentrations, causing some fibers to be inadequately stressed or even pulled out before the crack fully develops.

Secondly, the interfacial bond strength of the fibers is a critical factor influencing the bridging effect. In strain-softening specimens, swift crack propagation typically results in fiber failure predominantly by pull-out rather than rupture. Although computational models assume that fibers can provide their maximum bridging force, in practice, due to matrix interfacial defects, stress concentrations, and shear slip between the fiber and the matrix, many fibers fail to reach their theoretical maximum load capacity and are instead pulled out at lower stress levels. This interfacial effect further reduces the actual tensile strength, thereby contributing to the discrepancy between calculated and experimental values.

In summary, the rapid crack propagation, the brief duration of effective fiber bridging, and interfacial effects in strain-softening specimens often result in an actual tensile strength that is lower than the estimated value.

#### 3.4.3. Evaluation of Experimental and Estimated Tensile Strength

The actual cracking locations, predicted cracking locations, estimated tensile strength (*f_te_*), and theoretical tensile strength (*f_ta_*) were summarized alongside the experimental tensile strength (*f_t_*). The errors between the estimated tensile strength (*f_te_*), theoretical tensile strength (*f_ta_*), and experimental tensile strength (*f_t_*) were analyzed by examining experiment-estimation error (*E_exp_*) and theory-estimation error (*E_theo_*) in Table 10. *E_theo_* was introduced to minimize the influence mentioned in Section 3.3.2 that could affect the results. Specifically, *f_ta_*, calculated at the actual cracking location, serves as a benchmark to better evaluate the accuracy of *f_te_*, estimated at the predicted cracking locations.

As shown in Table 10, the accuracy of cracking location predictions plays a crucial role in determining the reliability of tensile strength estimation in UHPFRC. Moreover, an analysis of the fracture behavior of specimens reveals distinct trends in both error magnitudes and the effectiveness of cracking location predictions. In particular, strain-hardening specimens exhibit higher estimation accuracy and lower errors, with an average experiment-estimation error of 5.72% and an average theory-estimation error of 3.34%. While strain-softening specimens display greater deviations between predicted and actual crack locations, resulting in significantly higher errors (an average experiment-estimation error of 43.09% and an average theory-estimation error of 15.73%). These findings further indicate that successful cracking location predictions correspond to lower experiment-estimation errors and theory-estimation errors, while failed predictions lead to increased deviations—especially in strain-softening specimens.

**Figure 24:**
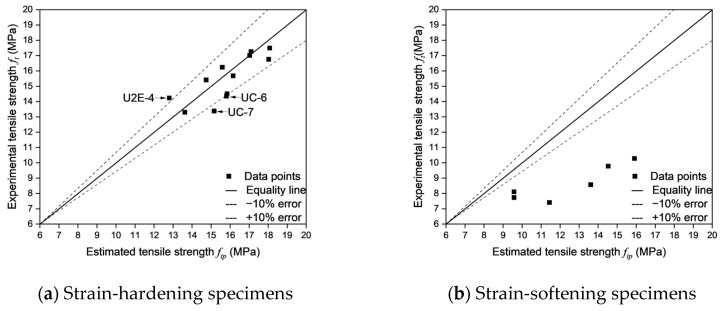
Experiment-estimation error.

**Figure 25:**
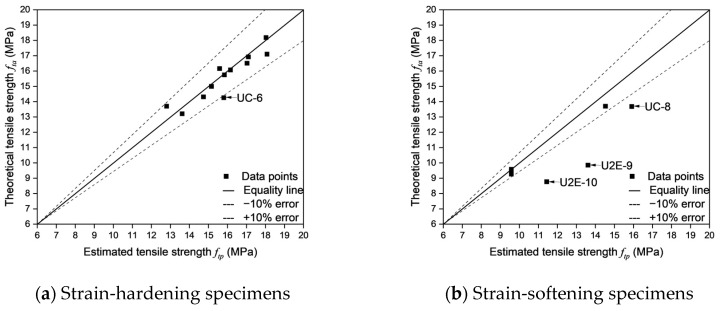
Theory-estimation error.

Strain-hardening specimens generally demonstrated more reliable cracking location predictions, with the majority of specimens classified as successful predictions. Such behavior can be attributed to a more uniform fiber distribution and enhanced fiber-matrix interaction, which in turn effectively controls crack propagation and facilitates the formation of multiple distributed cracks before ultimate failure. For instance, as shown in Figure 24a and Figure 25a, UC-4, UEE-1, and UEE-2, all of which had successful cracking location predictions, demonstrated low experiment-estimation errors and theory-estimation errors. Specifically, UC-4, with an actual cracking location of 45–55 and a predicted location of 40–60, had an experiment-estimation error of only −5.72% and a theory-estimation error of −3.39%, which suggests that the fiber distribution and orientation factors were sufficient to accurately predict tensile failure. Similarly, UEE-1, which has an actual cracking location of 10–18 (predicted cracking location: 10–20), showed an experiment-estimation error of 4.02% and a theory-estimation error of 3.57%, thereby reinforcing the notion that cracking prediction in strain-hardening specimens is highly reliable due to their controlled failure mechanism and effective fiber bridging.

However, not all strain-hardening specimens demonstrated perfect alignment between predicted and actual cracking locations. Specifically, certain specimens, including U2E-4 and UC-6, did not yield accurate cracking location predictions, which lead to larger deviations in errors despite their strain-hardening behavior. For example, U2E-4 had an actual cracking location of 53–63, while its predicted cracking location was 30–40, resulting in an experiment-estimation error of 10.06% and a theory-estimation error of −6.55%. Similarly, UC-6 exhibited an experiment-estimation error of −10.20% and a theory-estimation error of −10.92%, despite its classification as a strain-hardening specimen. These observations suggest that, even among strain-hardening specimens, localized fiber orientation inconsistencies may contribute to unpredictability in crack formation—although to a significantly lesser extent than in strain-softening specimens. The relatively low standard deviation in errors across strain-hardening specimens further indicates that these deviations are minor and do not fundamentally alter the predictability of tensile behavior.

In contrast, as shown in Figure 24b and Figure 25b, strain-softening specimens exhibited considerably larger deviations between predicted and actual cracking locations, which lead to substantially higher error values. Unlike strain-hardening specimens, which tend to develop multiple cracks and undergo progressive damage, strain-softening specimens are characterized by a single dominant crack. Consequently, their failure mechanisms are highly sensitive to local fiber depletion zones. This leads to greater unpredictability in crack formation and increased dispersion of errors. For example, U2E-9 and U2E-10, which did not yield accurate cracking location predictions, exhibited extremely high errors. Specimen U2E-9, which had an actual cracking location of (−12)–(−2) instead of the predicted cracking location (0–10), had an experiment-estimation error of −58.81% and a theory-estimation error of −38.17%—one of the highest values recorded in this study. Similarly, U2E-10 exhibited an experiment-estimation error of −54.41% and a theory-estimation error of −30.50%, further highlighting the challenges of predicting cracking locations in strain-softening specimens. These large discrepancies indicate that predictive models, which often assume a relatively uniform fiber orientation, are unable to accurately capture the actual failure behavior of strain-softening specimens.

The primary reason for higher error magnitudes observed in strain-softening specimens is the unpredictability of fiber alignment, which significantly influences cracking location prediction. Unlike strain-hardening specimens, in which cracks propagate in a more controlled manner owing to uniform fiber bridging, strain-softening specimens tend to exhibit more erratic crack formation mainly due to localized fiber depletion and misalignment. This observation is further supported by the fiber orientation factor (*μ*₀), which is consistently lower in strain-softening specimens, thereby indicating a less favorable fiber alignment for tensile load resistance. Additionally, the high standard deviation in errors among strain-softening specimens confirms that these deviations are not random but are inherently linked to the variability in fiber distribution.

A key observation across both strain-hardening and strain-softening specimens is the strong direct correlation between successful cracking location prediction and reduced error magnitudes. The analysis revealed that specimens with successful cracking location predictions exhibited significantly lower errors compared to those with failed predictions, regardless of their fracture behavior. The average experiment-estimation error for successfully predicted specimens was −11.85%, while for failed predictions, it was −39.42%, a nearly fourfold increase. Similarly, theory-estimation errors in failed predictions were six times higher (−18.78%) compared to successful predictions (−2.94%). This emphasizes that accurately predicting the cracking location is critical in reducing error magnitudes in both strain-hardening and strain-softening specimens. However, while strain-hardening specimens consistently demonstrated lower errors even in cases of failed predictions, strain-softening specimens exhibited severe deviations, indicating that failure prediction in these specimens remains a significant challenge.

While fracture behavior is the primary determinant of cracking location prediction accuracy and error magnitudes, the casting method also influences fiber orientation and, consequently, error variability. The experimental results indicate that casting from end to end (UEE) produced the lowest errors, likely due to its ability to ensure a more uniform fiber distribution across the entire specimen length. In contrast, the casting-from-two-ends (U2E) method also resulted in the highest errors, likely due to fiber alignment disruptions at the central region where flow streams meet. These findings reinforce the notion that fiber uniformity plays a critical role in cracking location prediction, and casting techniques that promote homogeneous fiber orientation should be prioritized to minimize prediction errors.

Overall, the results confirm that strain-hardening specimens exhibit lower deviations between predicted and actual cracking locations, leading to smaller errors and more reliable tensile strength estimations. Conversely, strain-softening specimens, due to their localized failure modes and erratic crack propagation, display significantly larger deviations and higher errors, making accurate estimations more challenging. The success or failure of cracking location prediction further amplifies these differences, with failed predictions corresponding to significantly higher error magnitudes. Finally, the role of casting methods in fiber orientation should not be overlooked, as more uniform casting approaches can reduce variability in crack formation and enhance the reliability of estimable models.

These findings emphasize the necessity for improved fiber distribution techniques and refined estimable models that account for stochastic fiber orientation effects, particularly in strain-softening UHPFRC specimens, where failure mechanisms remain highly variable. By incorporating enhanced fiber alignment strategies and optimizing casting methodologies, the predictability of crack formation and tensile response in UHPFRC can be significantly improved, leading to more accurate and reliable structural performance assessments.

While these findings clarify the limitations and strengths of the proposed method within different fracture behavior categories, it is also crucial to critically compare this approach against previous tensile strength estimation methodologies to further highlight its novelty and practical advantages.

Compared to previous tensile strength estimation frameworks such as SIA 2052-2016 [34] and the local distribution model by Shen et al. [33], the proposed method introduces two key advancements. Firstly, traditional models assume that critical cracking sections are known beforehand, requiring destructive sectioning to analyze fiber characteristics after specimen failure. This restricts their application in non-destructive testing and pre-emptive quality assurance. Secondly, by neglecting pre-crack localization and casting-induced fiber distribution variability, these models often exhibit reduced predictive reliability, especially in strain-softening UHPFRC.

In contrast, the present study leverages deep learning-based cracking location prediction combined with localized CT image fiber analysis, thereby enabling pre-failure strength estimation. This integrated methodology not only demonstrated high accuracy for strain-hardening specimens—with an average experimental-estimation error of 5.72%—but also provided insights into limitations in strain-softening specimens, thus presenting a more comprehensive and practical framework for UHPFRC tensile performance assessment.

## 4. Conclusions

This study developed and validated a novel integrated method combining deep learning-based cracking location prediction and CT image analysis for non-destructive tensile strength estimation of ultra-high-performance fiber-reinforced concrete (UHPFRC). The key findings are summarized as follows:

1. The casting method significantly influences fiber distribution and tensile performance. Casting techniques promoting uniform fiber flow, such as casting from center or end-to-end, result in improved fiber orientation and higher tensile strength, while casting from two ends leads to fiber misalignment and lower predictability.
2. Deep learning-based CT image analysis is effective for defect detection. Using a dataset of 1350 CT images, the YOLOv11 model achieved a mAP@0.5 of 0.87, demonstrating strong potential for non-destructive fiber distribution assessment in UHPFRC.
3. Cracking location prediction shows high accuracy for strain-hardening specimens. The proposed deep learning model successfully predicted cracking locations in strain-hardening UHPFRC specimens with a success rate of 73%, closely correlating with experimental observations.
4. Tensile strength estimation is accurate for strain-hardening specimens. The average experiment-estimation error was 5.72% and the theory-estimation error was 3.34%, confirming the method’s reliability. In contrast, strain-softening specimens exhibited higher errors due to more erratic crack propagation and local fiber depletion effects.
5. Localized fiber distribution defects introduce estimation errors. The uniform fiber distribution assumption in tensile strength estimation models can lead to significant errors when local fiber deficits exist, particularly near transition zones. Future refinements should address localized characterization.
6. Practical implications and future outlook. The proposed approach provides a reliable non-destructive framework for tensile strength evaluation in UHPFRC, with strong potential for quality control and early-stage defect detection. It can be applied during the design phase to verify tensile performance requirements, and in retrofitting to assess the quality of UHPFRC overlays or jacketing layers on existing RC components. For practical applications, the framework can be extended by applying localized CT imaging—or other high-resolution non-destructive techniques—to critical tensile zones of structural elements, such as beam ends, column bases, and anchorage regions where UHPFRC is cast or sprayed. By analyzing the in situ fiber distribution and estimating local tensile strength, the method enables engineers to verify whether performance targets are met, supporting design validation, construction quality assurance, and the acceptance of UHPFRC-enhanced components. Future work will focus on improving cracking prediction for strain-softening specimens and adapting the method to complex geometries and large-scale elements.

## Figures and Tables

**Table 2:** Mixture properties of UHPFRC.

Unit Amount		Reinforcing Fiber		Liquid-to-Powder Ratio
(kg/m^3^)		5% Fiber Content (kg/m^3^)		(%)
Standard blended powder	Special admixture	Steel fiber content	Steel wool content	Mixing liquid/standard blended powder
P	We	2.5 vol%	2.5 vol%	We/P
1840	290	196	196	16

**Table 3:** Specimen types and numbers.

Specimen Type	Casting Method	Numbers
U2E	Casting from two ends	10
UC	Casting from the center	8
U1E	Casting from one end	3
UEE	Casting from end to end	5
Total		26

**Table 4:** The hyperparameters used in YOLOv11.

Hyperparameter	Value
Batch size	4
Learning rate	0.001
Learning rate decay	0.9
Momentum	0.0005
Patience	100
Training epochs	600

**Table 5:** Modification of fiber counts.

**Specimen Groups**	**Modification Value**
U1E	+11
U2E	+11
UEE	+11
UC	+15

**Table 6:** Uniaxial tensile test results.

Specimen	Tensile Strength (MPa)	Main Cracking Area	Main Cracking Locations (mm)	Fracture Behavior
U1E-1	15.68	Within gauge length	5–18	s-h
U1E-2	16.59	Out of gauge length	(−5)–5	s-h
U1E-3	16.76	Within gauge length	15–30	s-h
Average	16.34			
U2E-1	12.61	Within gauge length	12–20	s-h
U2E-2	9.63	Out of gauge length	(−10)–(−2)	s-f
U2E-3	12.86	Out of gauge length	(−20)–0	s-f
U2E-4	14.23	Within gauge length	53–63	s-h
U2E-5	8.11	Within gauge length	30–36	s-f
U2E-6	9.86	Out of gauge length	(−15)–(−5)	s-f
U2E-7	9.78	Out of gauge length	(−2)–8	s-f
U2E-8	7.73	Within gauge length	45–56	s-f
U2E-9	8.57	Out of gauge length	(−12)–(−2)	s-f
U2E-10	7.41	Within gauge length	28–36	s-f
Average	10.11			
UC-1	15.65	Within gauge length	(−5)–12	s-h
UC-2	14.51	Out of gauge length	65–75	s-h
UC-3	14.90	Within gauge length	30–55	s-h
UC-4	17.49	Within gauge length	45–55	s-h
UC-5	17.26	Out of gauge length	(−10)–5	s-h
UC-6	14.35	Out of gauge length	(−15)–2	s-h
UC-7	13.38	Within gauge length	8–18	s-h
UC-8	10.28	Out of gauge length	(−15)–0	s-f
Average	14.73			
UEE-1	16.24	Within gauge length	10–18	s-h
UEE-2	17.01	Within gauge length	35–48	s-h
UEE-3	18.45	Within gauge length	10–18	s-h
UEE-4	13.32	Within gauge length	5–10	s-h
UEE-5	16.65	Out of gauge length	(−8)–(−4)	s-h
Average	16.33			

Note: “0” represents the starting point of the gauge length, and negative values indicate that the crack occurred outside the gauge length region.

**Table 7:** Cracking location prediction results.

Specimen	Actual Cracking Location	Predicted Cracking Location	Fracture Behavior	Overlap Rate (%)	Prediction Result	Specimen	Actual Cracking Location	Predicted Cracking Location	Fracture Behavior	Overlap Rate (%)	Prediction Result
U1E-1	5–18	10–20	s-h	53%	Successful	UEE-1	10––18	10–20	s-h	60%	Successful
U1E-2	(−5)–5	0–10	s-h	50%	Successful	UEE-2	35–48	40–50	s-h	53%	Successful
U1E-3	15–30	20–30	s-h	67%	Successful	UEE-3	10–18	10–20	s-h	80%	Successful
U2E-1	12–20	10–20	s-h	80%	Successful	UEE-4	5–10	0–10	s-h	50%	Successful
U2E-4	53–63	30–40	s-h	0%	Failed	UC-1	(−5)–12	0–10	s-h	83%	Successful
U2E-5	30–36	30–40	s-f	60%	Successful	UC-3	30–55	50–60	s-h	17%	Failed
U2E-7	(−2)–8	40–50	s-f	0%	Failed	UC-4	45–55	40–60	s-h	50%	Successful
U2E-8	45–56	50–60	s-f	40%	Failed	UC-5	(−10)–5	20–30	s-h	0%	Failed
U2E-9	(−12)–(−2)	0–10	s-f	×	×	UC-6	(−15)–2	10–20	s-h	0%	Failed
U2E-10	28–36	40–50	s-f	0%	Failed	UC-7	8–18	10–20	s-h	67%	Successful
						UC-8	(−15)–0	0–10	s-f	×	×

Note: “×” indicates that the actual crack location is outside the gauge length.

**Table 8:** Interfacial bond strength.

Specimens	Actual Cracking Location	Average Fiber Number	*μ*_0_	$\bar{\mu_{1}}$	*τ*_*f*_ (MPa)	*f_t_* (MPa)
UC-3	30–55	197	0.634	0.952	13.16	14.90
U2E-1	12–20	184	0.593	0.945	12.01	12.61
UEE-3	5–10	250	0.805	0.99	12.35	18.45
Average					12.50	

**Table 9:** Tensile strength estimation results at actual cracking locations.

Specimens	Actual Cracking Location	Average Fiber Count	Fracture Behavior	*μ*_0_	$\bar{\mu_{1}}$	*τ*_*f*_ (MPa)	*f_ta_* (MPa)	*f_t_* (MPa)	Error (%) $\frac{f_{t}-f_{ta}}{f_{t}}$
U1E-1	5–18	220	s-h	0.709	0.968	12.50	16.07	15.68	−2.52%
U1E-2	(−5)–5	199	s-h	0.641	0.953	12.50	14.31	15.41	7.11%
U1E-3	15–30	243	s-h	0.783	0.991	12.50	18.18	16.76	−8.45%
U2E-4	53–63	191	s-h	0.615	0.950	12.50	13.70	14.23	3.75%
U2E-5	30–36	138	s-f	0.444	0.890	12.50	9.27	8.11	−14.31%
U2E-7	(−2)–8	191	s-f	0.615	0.950	12.50	13.70	9.78	−40.04%
U2E-8	45–56	141	s-f	0.454	0.900	12.50	9.58	7.73	−23.91%
U2E-9	(−12)–(−2)	149	s-f	0.464	0.905	12.50	9.85	8.57	−14.93%
U2E-10	28–36	132	s-f	0.425	0.880	12.50	8.77	7.41	−18.32%
UC-1	(−5)–12	216	s-h	0.696	0.966	12.50	15.75	14.51	−8.54%
UC-4	45–55	231	s-h	0.744	0.981	12.50	17.10	17.49	2.20%
UC-5	(−10)–5	229	s-h	0.770	0.988	12.50	16.92	17.26	1.96%
UC-6	(−15)–2	202	s-h	0.731	0.978	12.50	14.26	14.35	0.65%
UC-7	8–18	207	s-h	0.667	0.960	12.50	15.00	13.38	−12.10%
UC-8	(−15)–0	191	s-f	0.615	0.949	12.50	13.68	10.28	−33.09%
UEE-1	10–18	221	s-h	0.712	0.969	12.50	16.16	16.24	0.47%
UEE-2	35–48	225	s-h	0.725	0.972	12.50	16.51	17.01	2.95%
UEE-4	5–10	185	s-h	0.596	0.946	12.50	13.21	13.3	0.68%

**Table 10:** Tensile strength estimation results at predicted cracking locations.

**Specimens**	**Actual Cracking Location**	Predicted Cracking Location	Cracking Location Prediction Result	Average Fiber Count at Predicted Cracking Location	Fracture Behavior	*μ*_0_	$\bar{\mu_{1}}$	*τ*_*f*_ (MPa)	*f_te_* (MPa)	*f_ta_* (MPa)	*f_t_* (MPa)	*E*_exp_ (%) $\frac{f_{t}-f_{te}}{f_{t}}$	*E*_theo_ (%) $\frac{f_{ta}-f_{te}}{f_{ta}}$
U1E-1	5–18	10–20	Successful	221	s-h	0.712	0.969	12.50	16.16	16.07	15.68	−3.09%	−0.56%
U1E-2	(−5)–5	0–10	Successful	204	s-h	0.657	0.957	12.50	14.74	14.31	15.41	4.37%	−2.94%
U1E-3	15–30	20–30	Successful	241	s-h	0.776	0.991	12.50	18.03	18.18	16.76	−7.56%	0.82%
U2E-4	53–63	30–40	Failed	180	s-h	0.580	0.942	12.50	12.80	13.70	14.23	10.06%	6.55%
U2E-5	30–36	30–40	Successful	141	s-f	0.454	0.900	12.50	9.58	9.27	8.11	−18.11%	−3.32%
U2E-7	(−6)–8	40–50	Failed	203	s-f	0.654	0.948	12.50	14.53	13.70	9.78	−48.53%	−6.06%
U2E-8	45–56	50–60	Successful	141	s-f	0.454	0.900	12.50	9.58	9.58	7.73	−23.91%	0.00%
U2E-9	(−12)–(−2)	0–10	×	190	s-f	0.612	0.949	12.50	13.61	9.85	8.57	−58.81%	−38.17%
U2E-10	28–36	40–50	Failed	163	s-f	0.525	0.930	12.50	11.44	8.77	7.41	−54.41%	−30.50%
UC-1	(−5)–12	0–10	Successful	217	s-h	0.699	0.967	12.50	15.84	15.75	14.51	−9.16%	−0.57%
UC-4	45–55	40–60	Successful	242	s-h	0.779	0.990	12.50	18.08	17.10	17.49	−3.39%	−5.72%
UC-5	(−10)–5	20–30	Failed	231	s-h	0.744	0.981	12.50	17.10	16.92	17.26	0.90%	−1.08%
UC-6	(−15)–2	10–20	Failed	218	s-h	0.702	0.961	12.50	15.81	14.26	14.35	−10.20%	−10.92%
UC-7	8–18	10–20	Successful	209	s-h	0.673	0.961	12.50	15.16	15.00	13.38	−13.31%	1.07%
UC-8	(−15)–0	0–10	×	218	s-f	0.702	0.967	12.50	15.91	13.68	10.28	−54.78%	16.30%
UEE-1	10–18	10–20	Successful	214	s-h	0.689	0.965	12.50	15.59	16.16	16.24	4.02%	3.57%
UEE-2	35–48	40–50	Successful	230	s-h	0.741	0.981	12.50	17.03	16.51	17.01	−0.12%	−3.17%
UEE-4	5–10	0–10	Successful	191	s-h	0.615	0.945	12.50	13.62	13.21	13.3	−2.44%	−3.13%

“×” means that the actual cracking locations are all outside the gauge length, making it impossible to calculate.

## Data Availability

The original contributions presented in this study are included in the article. Further inquiries can be directed to the corresponding author.
